# Mutations affecting the β-sheet at the extracellular opening of the intimin β-barrel domain lead to reduced protein levels and inefficient passenger secretion

**DOI:** 10.1042/BSR20253581

**Published:** 2026-02-06

**Authors:** Sai Priya Sarma Kandanur, Fabian A. Renschler, Lara Breuer, Monika S. Schütz, Jack C. Leo

**Affiliations:** 1 Section for Genetics and Evolutionary Biology, Department of Biosciences, University of Oslo, Olso, Norway; 2 Institute for Medical Microbiology and Hygiene, Interfaculty Institute of Microbiology and Infection Medicine (IMIT), Eberhard Karl University Tübingen, Tübingen, Germany; 3 German Centre for Infection Research (DZIF), Partner Site Tübingen, Tübingen, Germany; 4 Antimicrobial Resistance, Omics and Microbiota Group, Centre for Systems Health and Integrated Metabolic Research, Department of Biosciences, Nottingham Trent University, Nottingham, U.K.

**Keywords:** autotransporter, bacterial adhesion, enteropathogenic E. coli, expert on EPEC pathogenesis, gene regulation, intimin, inverse autotransporter, mutagenesis, pathogenesis, type 5 secretion system, Yersinia

## Abstract

Attaching and effacing pathogens, such as enteropathogenic and enterohaemorrhagic *Escherichia coli*, rely on an adhesin, intimin, for attachment to enterocytes and downstream effects leading to actin pedestal formation. Intimin belongs to the inverse autotransporter family (type 5e secretion systems), which secrete the extracellular adhesive domain or passenger via a hairpin intermediate. While many of the next steps in secretion are now understood, how the hairpin initially forms is not known. We sought to investigate this by making point mutations at several positions in the β-barrel domain of intimin, as this domain forms at least part of the secretion pore. We made the mutations in a wild-type background and in a stalled intermediate caught in the hairpin conformation, which allowed us to uncouple passenger secretion from hairpin formation. Surprisingly, most of the point mutations did not have an appreciable effect on hairpin formation or passenger secretion, and larger changes such as replacing the entire linker region with a flexible glycine–serine stretch showed only a modest reduction in passenger secretion. By contrast, mutations affecting a small β-sheet at the extracellular face of the intimin β-barrel between two extracellular loops and the C-terminus of the linker had a more pronounced effect on secretion, and abolishing this β-sheet prevented hairpin formation and led to complete loss of the wild-type protein. Our results show that the intimin β-barrel is remarkably tolerant to changes and that the β-sheet on the extracellular side of this domain plays a central role in passenger secretion and protein levels.

## Introduction

Intimin is a virulence factor of attaching and effacing pathogens, which include enteropathogenic and enterohaemorrhagic *Escherichia coli* (EPEC and EHEC, respectively). Intimin mediates the adhesion of these pathogens to enterocytes, which leads to a signal transduction cascade resulting in cytoskeletal rearrangements, the loss of microvilli and the formation of an actin pedestal around the bacteria [1]. Remarkably, the receptor for intimin is not a host protein but the translocated intimin receptor (Tir), which is translocated into host cells via a type 3 secretion system [2].

Intimin itself is an outer membrane protein with an extended extracellular region at the C-terminus including the Tir-binding domain [3], a transmembrane β-barrel domain [4], and a short periplasmic region contain a peptidoglycan-binding LysM domain [5]. The extracellular region or passenger consists of four immunoglobulin (Ig)-like domains and C-type lectin-like domain at the very C-terminus [3,6]. The passenger is connected to the β-barrel by a linker region that traverses the lumen of the β-barrel [4].

Over the past decade, it has become apparent that intimin is the prototype of a subclass of autotransporter proteins, termed type 5e secretion systems [7,8]. These are also referred to as inverse autotransporters because the domain order (N-terminal β-barrel and C-terminal passenger) is reversed when compared with classical (type 5a) autotransporters, which have an N-terminal passenger and a C-terminal β-barrel [9]. The mechanism of autotransport begins with the insertion of the intimin β-barrel into the outer membrane by the β-barrel assembly machinery (BAM) [7]. Probably concomitantly, a hairpin structure is formed between the linker and the N-terminal part of the passenger. The hairpin, which is an unfolded loop between the linker and the N-terminus of the passenger on the extracellular side of the β-barrel, positions the very N-terminus of the passenger at the cell surface, while the remainder of the passenger still resides in the periplasm [10]. The hairpin occupies the pore of the β-barrel, or more probably a hybrid pore formed by intimin and the central component of the BAM, BamA, as has been demonstrated for classical autotransporters [11,12]. The hairpin allows the first extracellular Ig-like domain D00 to fold at the extracellular surface, pulling more of the passenger through the pore until the next Ig-like domain D0 can fold, which in turn pulls through the next domain. Thus, the secretion is driven by sequential folding of Ig-like domains from the passenger N-terminus to the C-terminus, and the free energy for translocating the polypeptide through the pore is provided by the energy released when the domains in the passenger fold [13].

Although the mechanism of passenger secretion is now well understood, it is not known how the hairpin is formed in the first place. To address this issue, we undertook a mutagenesis study to try to pinpoint residues in the β-barrel domain that would be required for efficient hairpin formation and passenger secretion. To this end, we employed a stalled variant of intimin, IntHA453, where a double haemagglutinin (HA) tag was inserted into the D00 domain [10]. This prevents the folding of the D00 domain and traps the passenger at the hairpin stage [13]. Using this stalled variant allowed us to uncouple hairpin formation from passenger secretion The HA tag can still be detected at the cell surface in IntHA453, demonstrating the formation of the hairpin. We introduced a number of point mutations or larger-scale changes to conserved features in the β-barrel domain in both the wild-type (WT) intimin and IntHA453 backgrounds and examined surface exposure of either the C-terminus of the passenger (in the WT background) or the double HA tag as a proxy for hairpin formation in the IntHA453 background. We found that hairpin formation and passenger secretion are remarkably robust, and most alterations had little to no effect. However, changes to the small β-sheet at the extracellular surface of the β-barrel which forms between the linker and two extracellular loops caused secretion defects or loss of the entire protein. Our results suggest this β-sheet is crucial for maintaining intimin protein levels and secretion.

## Results

### Identification of interactions in the intimin β-barrel domain

**Figure 1:**
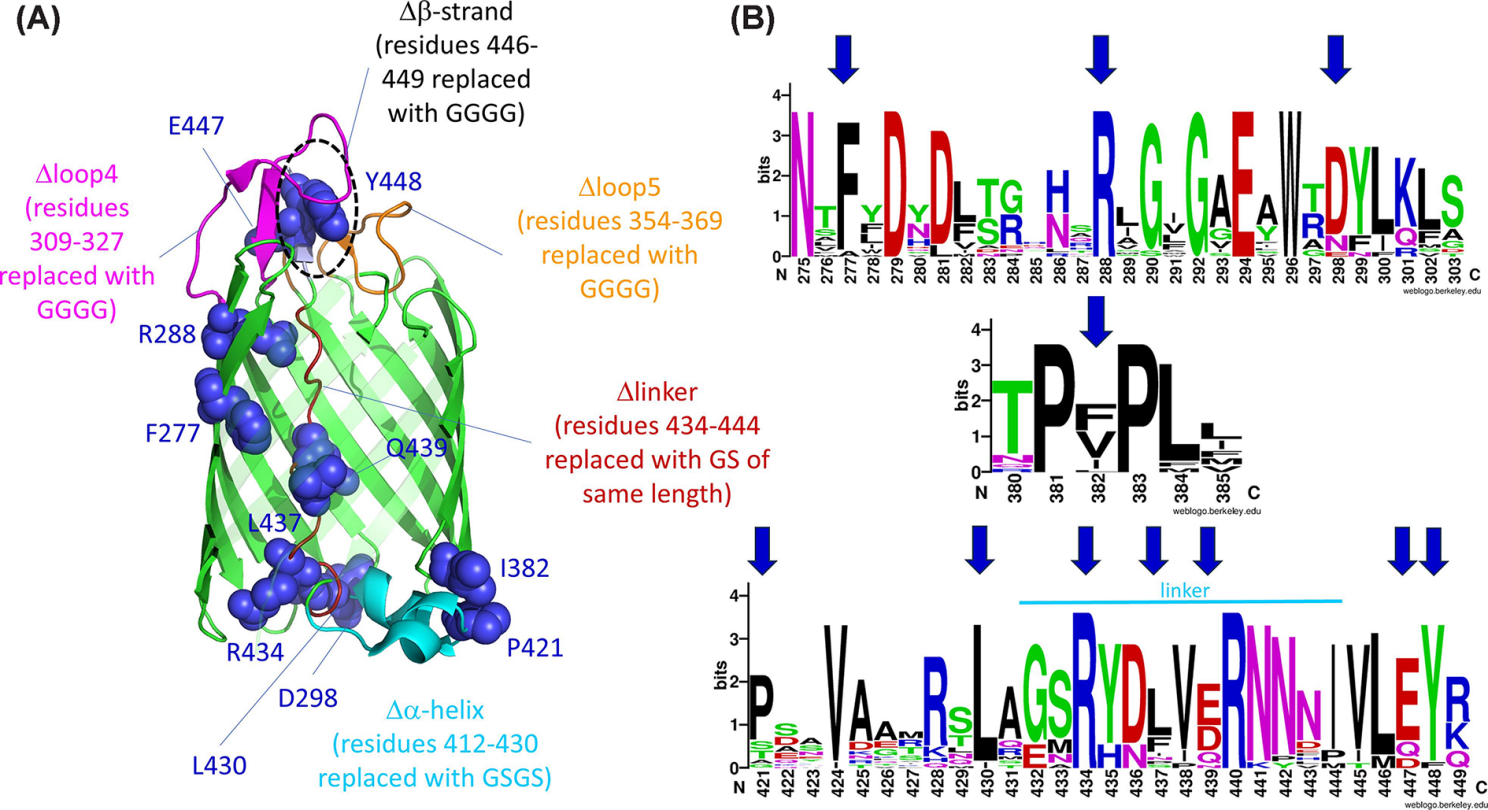
Residues and regions in the intimin β-barrel domain targeted for mutation (**A**) The residues targeted for point mutation in the intimin β-barrel are highlighted as blue spheres. Larger mutations are highlighted in different colours (Δα-helix, cyan; Δlinker, dark red; Δloop4, magenta; Δloop5, orange), and the Δβ-strand is encircled in black. Detailed interactions of the targeted residues and regions are given in Supplementary Figures S1–3. The figure was prepared using PyMOL (Schroedinger). (**B**) Conservation of the targeted residues in inverse autotranporters. Sequence logos based on an alignment of 20 inverse autotransporter sequences. Targeted residues are highlighted with arrows.

To investigate hairpin formation, we decided to target interactions between residues in the linker and residues lining the lumen of the β-barrel or in the surface loops. These were based on the existing crystal structure of the intimin β-barrel [4]. We reasoned that such interactions would be needed to stabilize the linker and may contribute to hairpin formation. We focussed on three regions of the β-barrel: the periplasmic face, with an α-helical turn that plugs the bottom of the β-barrel, the linker itself as it traverses the pore of the β-barrel, and the small antiparallel β-sheet formed between two extracellular loops (loops 4 and 5) and the linker as it exits the pore (Figure 1). We made changes to these regions by site-directed mutagenesis, targeting conserved residues (Figure 1B).

In the first region, we targeted residues D298, I382, P421, and L430. D298, located on periplasmic turn 3 of the β-barrel and facing the lumen, makes a hydrogen bond with the amide nitrogen of L430, potentially stabilizing the N-terminus of the linker (Supplementary Figure S1). Changing this residue to alanine would disrupt this interaction. I382, at the C-terminal end of β-strand 14, forms the core of a hydrophobic cluster involving residues L384, V385, I419, and P421 (Supplementary Figure S1). The latter two are located on the α-helical turn. We reasoned that disrupting this hydrophobic cluster by changing I382 to a charged residue (aspartate) would destabilize the α-helical turn. P421 is located between the two short α-helices in the turn (Supplementary Figure S1). As a known secondary structure breaker, the proline at this position may be important to introduce the kink between the α-helices, so changing it to alanine may have an impact on the structure of this region. L430 is involved in a hydrophobic interaction with F266 on β-strand 4 (Supplementary Figure S1). This interaction might be important in positioning the N-terminus of the linker, and changing L430 to alanine should disrupt this interaction. In addition to these point mutations, we changed the entire α-helical turn (referred to as Δα-helix, residues 412–430) with a flexible (serine-glycine)_2_ stretch, which is shorter than the α-helical turn but long enough to allow the linker to form in the correct position.

In the linker, we changed residues F277, R288, R434, L437, and Q439, all to alanine. F277 is located on β-strand 4 and packs against V438, located in the linker (Supplementary Figure S2); substituting F277 with alanine would abrogate this interaction. R288, on β-strand 6, is part of a hydrogen bonding network involving N441 and N443 at the C-terminus of the linker and Y353 on β-strand 9 (Supplementary Figure S2). This network would be lost upon exchanging R288 (completely conserved in inverse autotransporters, Figure 1B) to alanine. R434, located in the linker, is also completely conserved and interacts with the amide of E238 in intracellular turn 1 and has a cation-pi interaction with F266 on β-strand 4 (Supplementary Figure S1). L437 packs between L231 on β-strand 2 and the β-methylene groups of E214 and D229, on β-strands 1 and 2, respectively (Supplementary Figure S2); a substitution of L437 with alanine would disrupt these interactions. Q439 forms hydrogen bonds with K301 and the carbonyl group of S303 on β-strand 7. As for the α-helical turn, we assessed the global role of the linker by replacing all residues (434–444) with a glycine-serine stretch of the same length (referred to as Δlinker).

**Figure 2:**
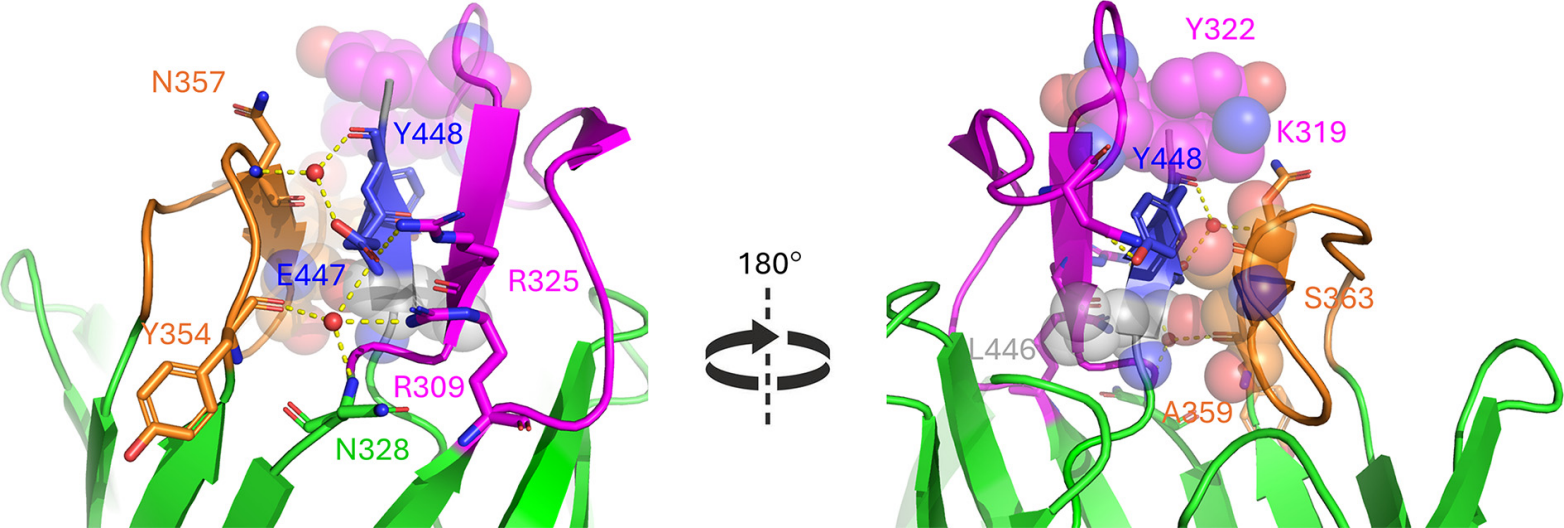
Residues and interactions targeted at the extracellular region of the intimin β-barrel Mutated residues are shown in blue, in either space-filling or stick representation; interacting residues are shown according to the colour scheme in Figure 1, and hydrogen or ionic bonds in dashed yellow lines. The left panel centres on E447, in the β-strand at the C-terminal end of the linker, which is at the centre of an interaction network between several residues in the neighbouring loops. The right panel centres on Y448 on the opposite side of the β-sheet, which is at the core of a hydrophobic cluster involving residues from loop 4 (N318, K319, Y322), loop 5 (A359, S363) and the linker (L446). Y448 is shown in stick representation, whereas other residues taking part in the hydrophobic cluster are shown as partly transparent spheres. These interactions are also highlighted in Supplementary Figure S3. The figures were prepared in PyMOL (Schroedinger).

At the extracellular face of the β-barrel (Figure 2), we replaced E447 with alanine and Y448 with aspartate. Both residues are in the β-strand at the C-terminal tip of the linker. E447 makes a salt bridge with R325 and contacts R309, N328, N357, and Y354 through a bridging water molecule (Supplementary Figure S3). The highly conserved Y448 (Figure 1B) is at the centre of a hydrophobic cluster along with K319, Y322, A359, S363, and L446 that is expected to be disrupted in the Y448D mutation. In addition, Y448 forms a hydrogen bond to N318 on loop 4 (Supplementary Figure S3). We also changed this short β-strand (residues 446–449) entirely into glycines (Δβ-strand). In addition, we deleted extracellular loop 4 (Δloop4) by replacing residues 309–369 with four glycines, and loop 5 (Δloop5) by replacing residues 354–369 by four glycines. These four remaining, flexible residues would be able to connect to the adjacent β-strand but this results in fully deleting the extracellular loops.

In order to assess the effects of our mutations on the final folded state of intimin, we generated models using Alphafold 3 (AF) [14]. To compare overall fold quality, we chose the AF predicted template modelling score (pTM). The highest pTM scores of 0.562 ± 0.007 was observed for the WT protein and all other mutants tested reached similar high scores. The only two mutants with pTM scores below 0.5 are the IntHA453 variant with 0.404 ± 0.008 and the Int Δloop4 mutant 0.482 ± 0.007, indicating overall problems of AF to achieve high confidence predictions for these variants. As those two intimin variants also displayed one of the most pronounced phenotypes in comparison with the WT protein (see below), we were not surprised. Furthermore, we aligned all models belonging to one mutant in PyMOL and use the resulting RMSD between the structures as an indicator for overall similarity in domain arrangement predictions of that mutant. Unsurprisingly, the only prediction which did not return very similar models with RMSDs below 4.5 Å was the IntHA453 variant with an RMSD of 22.325 ± 1.560 Å. The presence of the HA-tag yielded to a loss in coherence of the extracellular domains and the β-barrel domain but were unable to capture the stalled hairpin as suggested from previous research [10,13] This is not surprising, given the scope of AF to only model the final structure of a protein and not its biogenesis. As AF predictions are usually not suitable to resolve the effects of mutations on the fold of a protein due to its inherent properties and due to the lack of physical and chemical knowledge as a result of its training, as reported recently [15], we opted to focus on the pLDDT scores for evaluation the predicted effects of our mutations on the intimin folding. The presence of the HA tag clearly influences the predictions of intimin across its whole structure and the most pronounced ΔpLDDT changes are observed next to the HA tag in the connector which is highly destabilized with an ΔpLDDT = −18.140. By contrast, the effects of our mutants usually do not translate across the whole intimin structure and are more localized. Also, the effects of single amino acid substitutions are less pronounced if present at all. For example, the Δβ-strand mutation destabilizes the β-strand itself (ΔpLDDT = −8.984) and the directly adjacent connector (ΔpLDDT = −7.882), loop4 (ΔpLDDT = −6.130), and loop5 (ΔpLDDT = −3.134) but fails to induce changes within the directly adjacent linker whereas the Y448D mutation within the β-strand only leads to minor destabilizations of the β-strand (ΔpLDDT = −2.198), loop 4 (ΔpLDDT = −1.230), and loop 5 (ΔpLDDT = −1.606). This again, is in line with the severity of the experimental phenotypes. The exception to this are the mutations replacing parts of the molecule with G or G/S stretches (Δβ-strand, Δloop4, Δloop5, Δlinker, Δα-helix), which have pronounced local effects with the largest pLDDT change observed in chase of the Δα-helix mutation of ΔpLDDT = −39.108. However, this large local impact does not translate into the extracellular site in a negative manner but seems to slightly stabilize loop 4 and the D00 domain (ΔpLDDT = 0.708 and ΔpLDDT = 1.960, respectively). A full discussion of these predictions is provided in the Supplementary Material (Supplematary Information and Supplementary Figures S4−6).

### Mutations at the periplasmic face of the intimin β-barrel do not affect hairpin formation or passenger secretion

**Figure 3:**
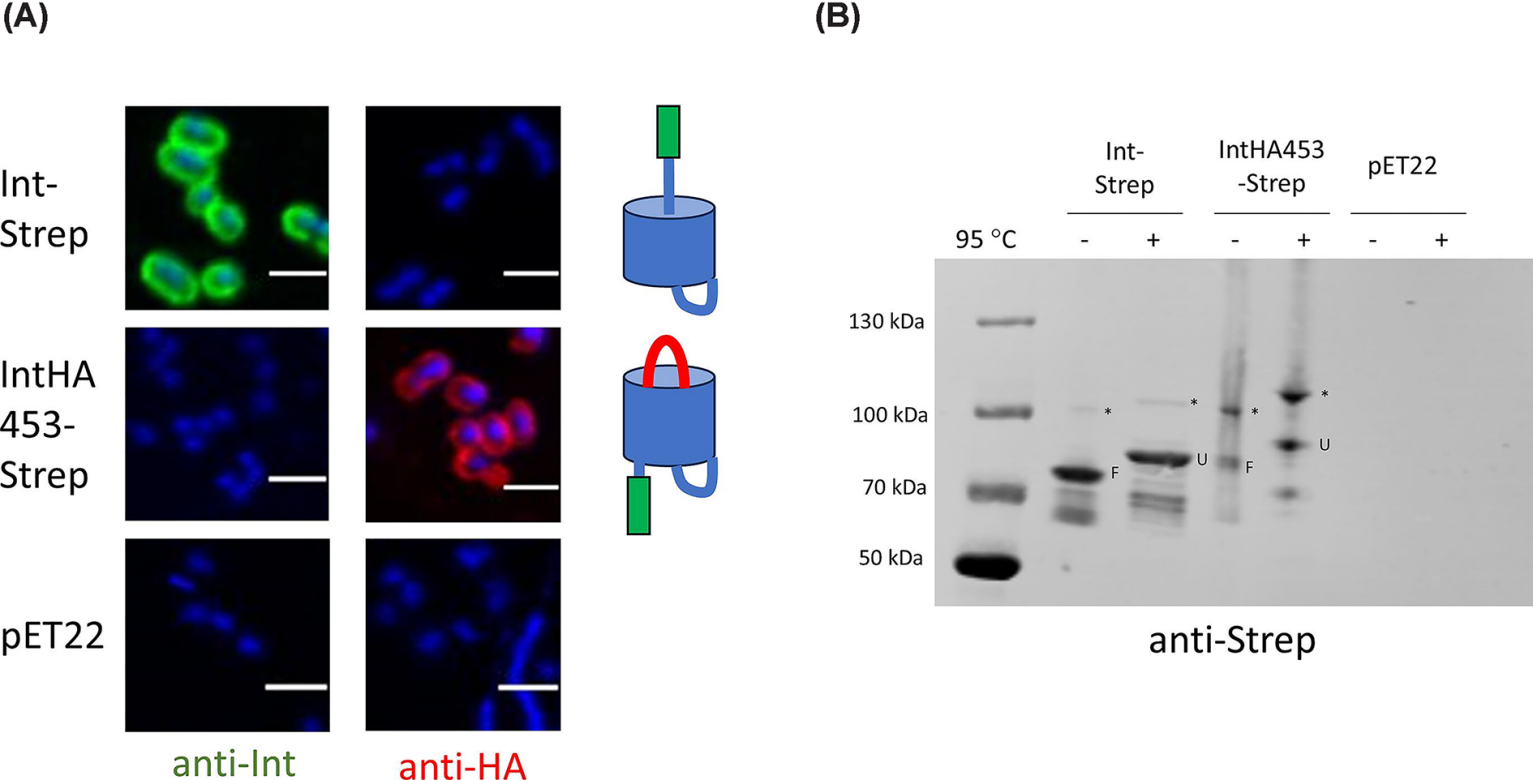
Surface exposure of intimin variants in autoinduced cultures (**A**) Confocal microscopy of cultures stained with an anti-intimin (anti-Int, left) or anti-HA tag (anti-HA, right) antibody. Cells were counterstained with DAPI. The schematics on the right of the microscopy images show the expected conformation of the proteins. pET22 is the vector control. The scale bar is 2 μm. (**B**) Western blot of outer membrane samples with intimin variants using an anti-Strep tag primary antibody. Samples were split in half and incubated at either room temperature or 95°C for 10 min and run in a 4–12% Novex gel to display the heat shift typical of β-barrel proteins before transferring to a nitrocellulose membrane. Molecular weight standards are notated on the left. F = folded, U = unfolded, * = band of unknown origin.

We introduced the mutations discussed above into both WT intimin (Int-Strep) and Intimin HA453 (IntHA453-Strep), both including a C-terminal StrepII tag. We tested the surface exposure of the passenger by immunofluorescence microscopy using an antibody against the C-terminus of intimin: as expected, cells producing Int-Strep were stained, whereas cells producing IntHA453-Strep did not stain with this antibody (Figure 3A). However, an anti-HA antibody did stain IntHA453-Strep, demonstrating that the double HA tag is surface-exposed and the hairpin is formed, as shown before [10]. A western blot with an anti-Strep tag antibody demonstrates that the Int-HA453-HA protein is intact, though expressed at lower levels than WT Int-Strep (Figure 3B). Both proteins exhibit the characteristic gel shift upon boiling that indicates unfolding of the β-barrel, which shows both proteins are correctly folded and inserted into the outer membrane. The blot also shows an additional, higher molecular-weight band (indicated by an asterisk in Figure 3B), which has been seen before [8,10,13], but the nature of which remains elusive. This additional band is also present in a blot of blue native (BN) –PAGE of WT intimin (Supplementary Figure S7). It is, however, clear that this band is not due to incorrect disulphide formation in the C-terminal domain of intimin, as addition of a reducing agent had no effect on this band (Supplementary Figure S8). Similarly, probing with the anti-Int antibody shows that IntHA453-Strep is produced at lower levels but that it is intact (Supplementary Figure S9). The lower expression of IntHA453-Strep seen in this study is possibly due to the autoinduction conditions used. With a longer expression time, the association of IntHA453 with the BAM complex [10] might lead to a reduction in outer membrane insertion efficiency over time. The empty vector control (pET22) failed to stain with either the anti-Intimin or the anti-HA antibody (Figure 3A).

**Figure 4:**
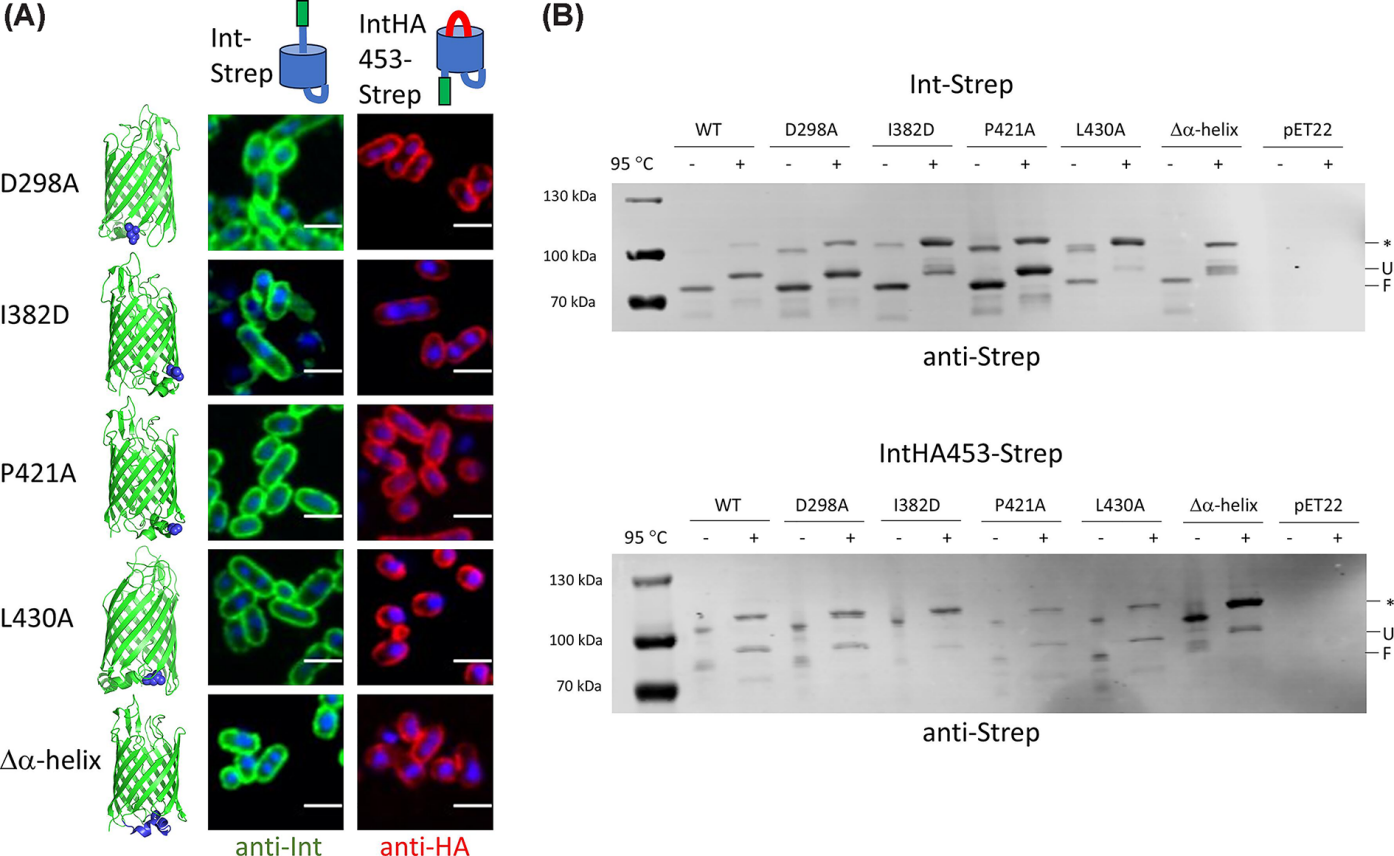
Effect of mutations on the periplasmic face of the intimin β-barrel (**A**) Confocal microscopy of intimin mutants in both the Int-Strep (left, probed with an anti-Strep antibody, coloured in green) and IntHA453-Strep (right, probed with an anti-HA antibody, coloured in red) backgrounds. Cells were counterstained with DAPI. Structures on the left highlight the position of the mutation. Schematics at the top show the expected conformation of the proteins. The scale bar denotes 2 μm. (**B**) Western blots of outer membrane samples with intimin variants using an anti-Strep tag primary antibody. The upper blot is in the Int-Strep background and the lower blot in the IntHA453-Strep background. Samples were split in half and incubated at either room temperature or 95°C for 10 min and run in a 4–12% Novex gel to display the heat shift typical of β-barrel proteins before transferring to a nitrocellulose membrane. pET22 is the empty vector. Molecular weight standards are notated on the left of the gels. F = folded, U = unfolded, * = band of unknown origin.

**Figure 5:**
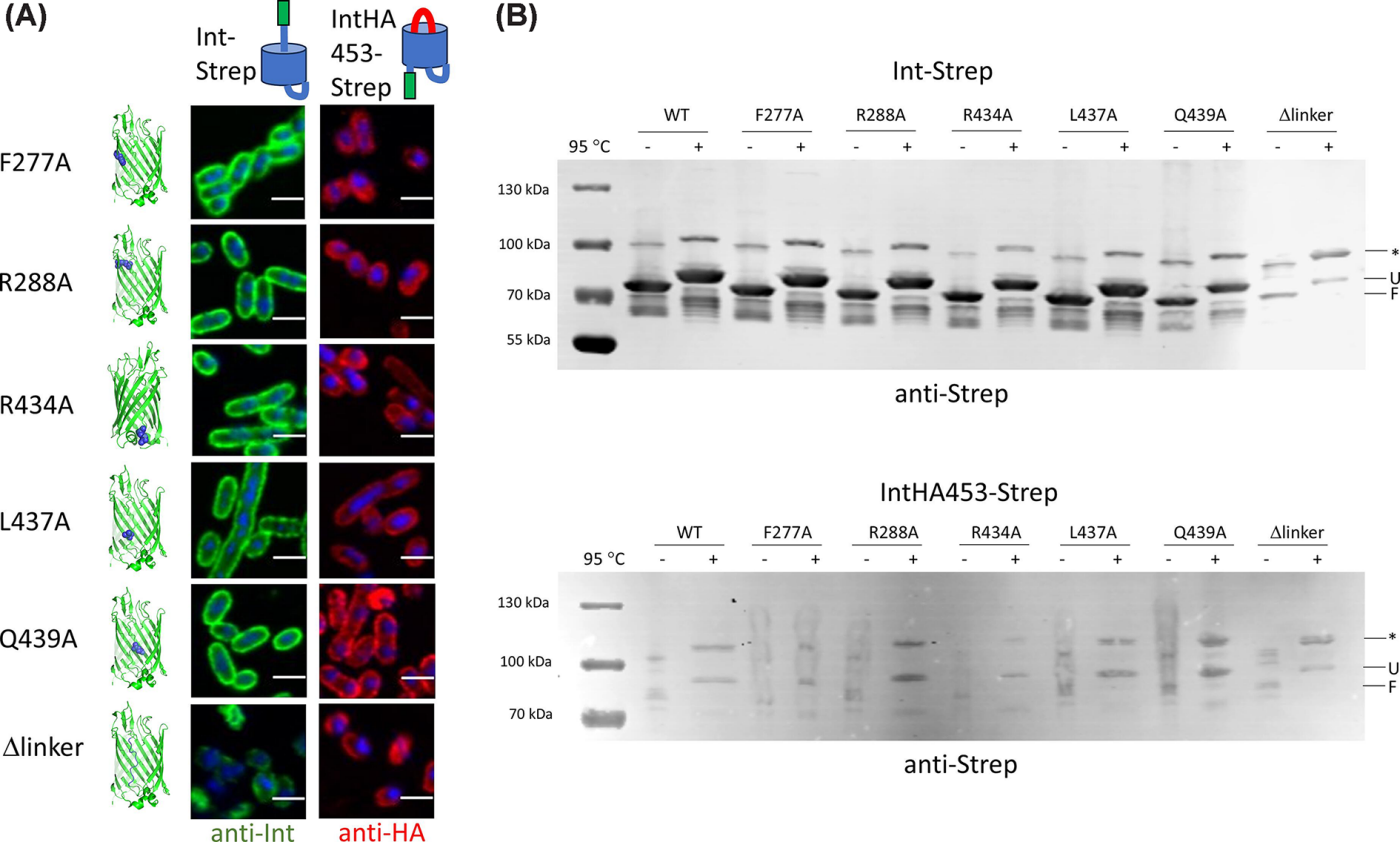
Effect of mutations on the linker region of intimin (**A**) Confocal microscopy of intimin mutants in both the Int-Strep (left, probed with an anti-Strep antibody, coloured in green) and IntHA453-Strep (right, probed with an anti-HA antibody, coloured in red) backgrounds. Cells were counterstained with DAPI. Structures on the left highlight the position of the mutation. Schematics at the top show the expected conformation of the proteins. The scale bare is 2 μm. (**B**) Western blots of outer membrane samples with intimin variants using an anti-Strep tag primary antibody. The upper blot is in the Int-Strep background and the lower blot in the IntHA453-Strep background. Samples were split in half and incubated at either room temperature or 95°C for 10 min to display the heat shift typical of β-barrel proteins and run in a 4–12% Novex gel to display the heat shift typical of β-barrel proteins before transferring to a nitrocellulose membrane. Molecular weight standards are notated on the left of the gels. F = folded, U = unfolded, * = band of unknown origin.

We then proceeded to examine the surface exposure of the various mutants, beginning with those on the periplasmic face of intimin. Remarkably, none of the mutations had a large effect on surface exposure of either the passenger in the Int-Strep background, or of the double HA tag in the IntHA453-Strep background (Figure 4A). All the proteins were produced, as verified by western blot, and all the mutations in the Int-Strep background exhibited a heat shift (Figure 4B), which suggests all the proteins were correctly folded and inserted into the outer membrane [16]. Interestingly, upon heating, some mutants (I382D and L430A) seemed to preferentially populate the higher molecular-weight band (denoted by an asterisk in Figures 3-5). Similarly to the WT IntHA453-Strep, mutants in this background were produced at lower levels than Int-Strep (Figure 4B). An exception to this seemed to be the Δα-helix mutant, but this (like the I382D mutant) appeared to preferentially populate the higher molecular weight bands in the gel. Nonetheless, the mutant proteins all exhibited a heat shift (Figure 4B), and they were produced at high enough levels to be easily detected with the anti-HA antibody in immunofluorescence microscopy (Figure 4A). These results suggest that interactions at the periplasmic face of the intimin β-barrel are not important for efficient hairpin formation and passenger secretion.

### Substituting the entire linker reduces secretion and hairpin formation

We then investigated whether interactions between the linker and the luminal face of the β-barrel affect hairpin formation and secretion. Similarly to our findings regarding the α-helical turn, individual point mutations in this region did not have an impact on passenger secretion or hairpin formation (Figure 5A). Only when the entire linker was replaced by a flexible sequence made of GS repeats did we observe an effect. Surface exposure of the passenger was reduced in the Int-Strep Δlinker variant in both backgrounds. Consistent with this observation, the amount of protein was reduced for Int-Strep Δlinker in the western blot, though the heat shift was still evident (Figure 5B, upper panel). The reduction was also not due to degradation by the periplasmic protease DegP, which is known to play a role in inverse autotransporter quality control [7,17–19] (Supplementary Figure S10). All the other Int-Strep mutants behaved like the WT in the western blot (Figure 5B). Similarly to the previous set of mutants in the IntHA453, protein levels were reduced compared to WT Int-Strep, but all mutants displayed a heat shift (Figure 5B, lower panel).

### Changes to the β-sheet at the extracellular face of the β-barrel affect secretion and intimin protein levels

**Figure 6:**
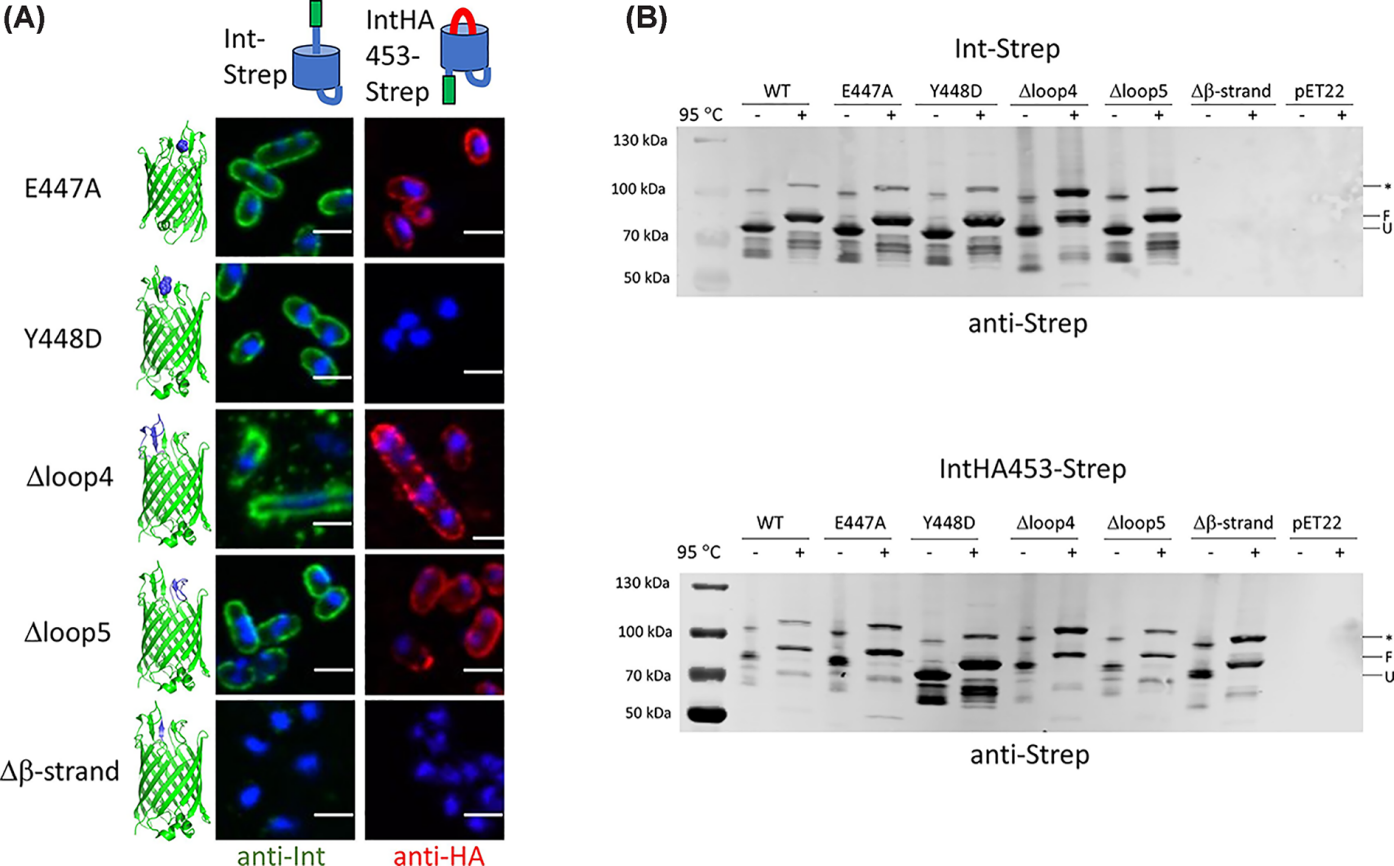
Effect of mutations on extracellular face of the intimin β-barrel (**A**) Confocal microscopy of intimin mutants in both the Int-Strep (left, probed with an anti-Strep antibody, coloured in green) and IntHA453-Strep (right, probed with an anti-HA antibody, coloured in red) backgrounds. Cells were counterstained with DAPI. Structures on the left highlight the position of the mutation. Schematics at the top show the expected conformation of the proteins. The scale bar denotes 2 μm. (**B**) Western blots of outer membrane samples with intimin variants using an anti-Strep tag primary antibody. The upper blot is in the Int-Strep background and the lower blot in the IntHA453-Strep background. Samples were split in half and incubated at either room temperature or 95°C for 10 min and run in a 4–12% Novex gel to display the heat shift typical of β-barrel proteins before transferring to a nitrocellulose membrane. pET22 is the empty vector. Molecular weight standards are notated on the left of the gels. F = folded, U = unfolded, * = band of unknown origin.

We examined the effect of changes in the β-sheet at the extracellular side of the β-barrel by making changes to residues involved in interactions presumed to stabilize the β-sheet. Though the E447A mutation did not have an effect on either hairpin formation or passenger secretion, the Y448D mutation did have a clear effect on hairpin formation, and the IntHA453-Strep Y448D-expressing cells failed to stain with the HA-antibody (Figure 6A). This was despite the protein being produced and exhibiting a normal heat shift (Figure 6B). Surprisingly, replacing the extracellular loops 4 and 5 with a shorter stretch of glycines did not have a large effect on either passenger secretion or hairpin formation (Figure 6A), though removing the longer loop 4 did lead to an increase in the higher molecular-weight band in the western blot (Figure 6B). By contrast, replacing the β-strand at the C-terminus of the linker with glycines had a dramatic effect, completely abolishing both hairpin formation and passenger secretion (Figure 6A). This was due to no protein being present in the outer membrane for the Int-Strep Δβ-strand mutant (Figure 6B), which we interpret to mean that the protein was destabilized and completely degraded. No protein was detected in whole cell extracts either, even when the periplasmic protease DegP was absent, demonstrating the importance of this β-strand for protein stability (Supplementary Figure S10). However, in the IntHA453-Strep variant, neither the Y448D nor the Δβ-strand mutation resulted in loss of the protein, though the HA tag could not be detected at the cell surface, suggesting the hairpin was not correctly formed (Figure 6B).

### Mutants affecting the linker and extracellular β-sheet have reduced surface exposure of the intimin passenger

**Figure 7:**
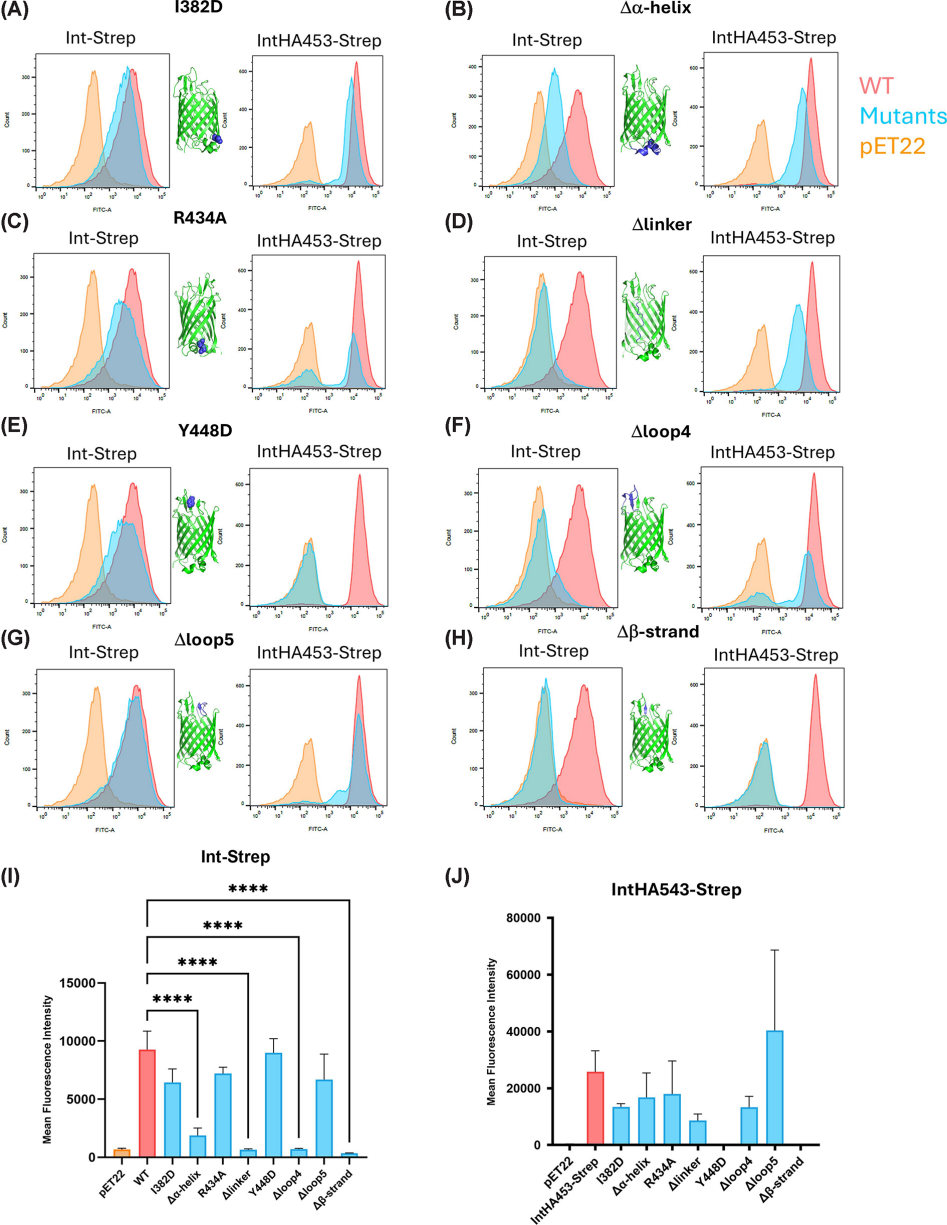
Flow cytometry analysis of selected intimin mutants The data for the mutants (in both the Int-Strep and IntHA453-Strep backgrounds) are shown by histograms in light blue, the WT is shown in red and the empty vector (pET22) in orange. Bacteria were stained with an anti-Strep primary antibody (for the Int-Strep variants) or an anti-HA antibody (for the IntHA453-Strep variants). Representative histograms (*N*=2–3) are shown. (**A**) I348D mutant. (**B**) Δα-helix mutant. (**C**) R434A mutant. (**D**) Δlinker mutant. (**E**) Y448D mutant. (**F**) Δloop4 mutant. (**G**) Δloop5 mutant. (**H**) Δβ-strand mutant. (**I**) Mean fluorescence intensity of cytometry experiments in the Int-Strep background. (**J**) Mean fluorescence intensity of cytometry experiments in the IntHA453-Strep background. ****P<0.001 (one-way ANOVA with Dunnett’s post hoc test; non-significant differences are not indicated).

To quantify the amount of protein exposed at the surface of the intimin mutants demonstrating reduced staining in immunofluorescence microscopy, we performed flow cytometry. Cells expressing Int-Strep or IntHA453-Strep stained well with an anti-Strep or anti-HA antibody, respectively (Figure 7). We tested the point mutants Y448D and the mutants affecting larger regions, Δα-helix, Δlinker, Δloop4, Δloop5, and Δβ-strand. In addition, we included two point mutants that had no effect in our microscopy analysis (I382D and R434A) from the periplasmic face of the lumen of the intimin β-barrel, respectively, as controls. The results are largely consistent with what we observed in the fluorescence microscopy: mutants I382D and Δloop5 did not have an appreciably affect (Figure 7A,G) in either the Int-Strep or the IntHA543-Strep backgrounds, whereas deleting the β-strand essentially abolished fluorescence in both (Figure 7H). The Δα-helix variant showed a modest reduction in fluorescence in both cases (Figure 7B), but the Δlinker mutant lost almost all fluorescence in the Int-Strep background, with only a slight reduction in the IntHA453-Strep background (Figure 7D). Interestingly, the R434A mutation resulted in a bimodal distribution in the IntHA453-Strep background, suggesting a slight defect in hairpin formation, but passenger secretion was not impaired (Figure 7C). This is consistent with there being no significant changes caused by this mutant in the WT background (Figure 7C). R434 may help to stabilize the hairpin, as it is located at the N-terminal end of the linker, at the juncture with the α-helical loop, which could explain the bimodal distribution through backsliding of the hairpin into the periplasm. For the Δloop4 variant, fluorescence was significantly reduced in the Int-Strep background, whereas a bimodal distribution was again seen in the IntHA453 background, which might also be due to some backsliding as loop 4 participates in the β-sheet at the extracellular opening of the β-barrel. Quantification of the mean fluorescence intensities (Figure 7I and J) showed that the Δα-helix, Δloop4, and Δβ-strand were all significantly reduced compared with the WT. By contrast, in the IntHA453-Strep set, none of the variants reached significance in a formal statistical test, but the reduction in fluorescence for the Y448D and Δβ-strand variants is clear. The empty vector control was significant in both cases (*P*<0.05, not shown in Figure 7).

We reasoned that the reduction of protein levels in the Δβ-strand variants, Δlinker variants and IntHA453-Strep Y448D might be due to loss of the plasmid if expression of these constructs were toxic to the cell. We therefore checked plasmid retention levels by plating dilutions of autoinduced cultures on both LB without selection and LB + ampicillin (Supplementary Figure S11). All overnight cultures expressing intimin displayed plasmid loss, but this was at a similar level and is not enough to explain the complete absence of the protein in the Δβ-strand variants and IntHA453-Strep Y448D. Therefore, any reduction in protein production levels for these variants must be due to either the more rapid degradation of the resulting proteins or inefficient membrane integration.

To check whether these mutations might make the proteins more susceptible to proteolysis, we performed a protease shaving assay. For this, we moved the StrepII tag from the C-terminus to the N-terminus of mutants in the WT background, after the signal peptide cleavage site, to protect the tag. However, these experiments proved inconclusive, as all intimin variants lost the signal in a western blot after protease treatment (Supplementary Figure S12).

### Mutations abolishing secretion also prevent attachment to host cells

**Figure 8:**
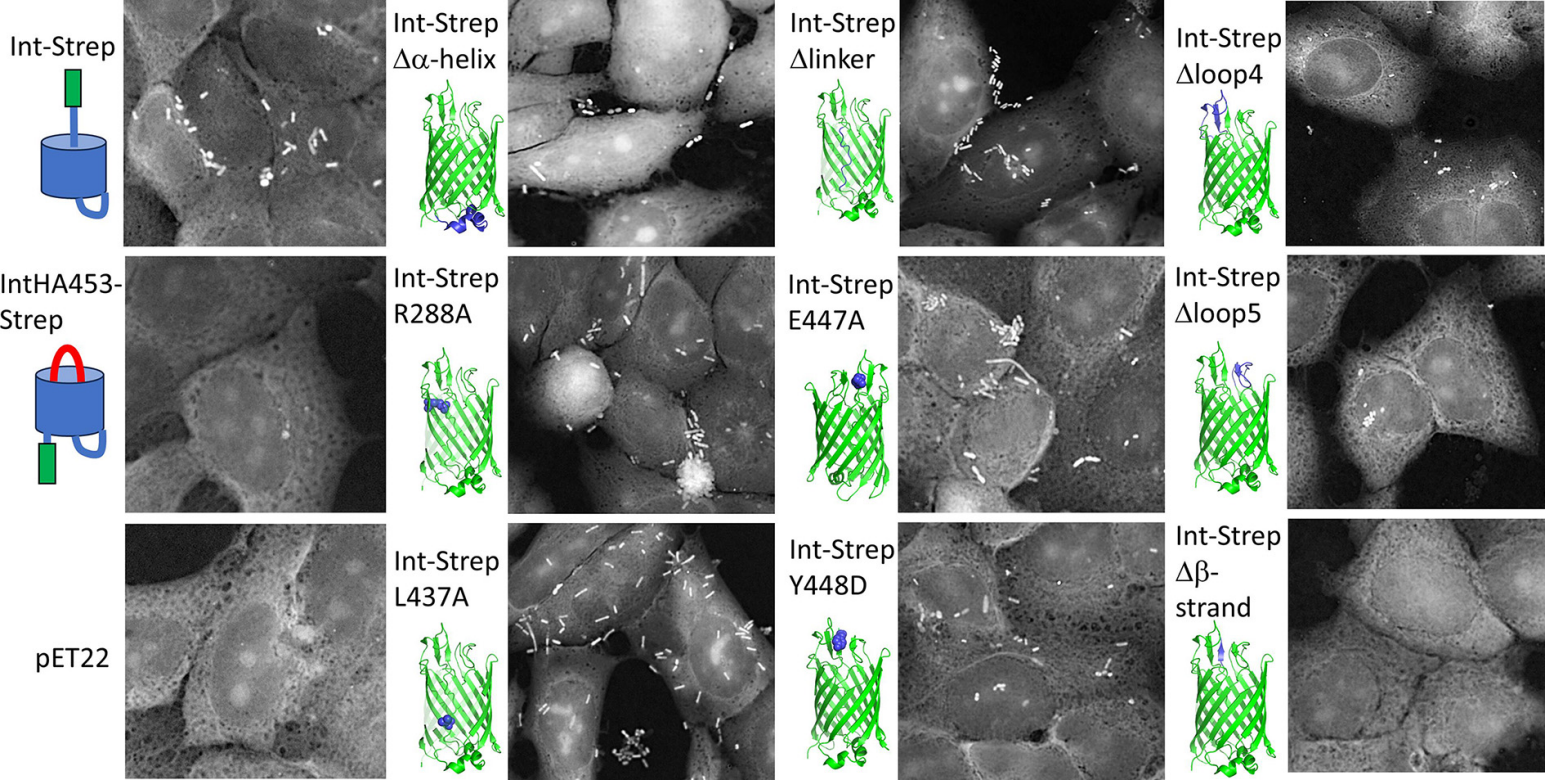
Binding of intimin variants to Tir-displaying HeLa cells Cells were primed with EPEC lacking intimin, after which BL21ΔF cells producing intimin variants were allowed to adhere. Non-adherent cells were washed off and the cells were stained with fuchsin. The images are shown in negative to highlight the bacteria. Schematics show the expected conformations of control experiments, and the structures highlight the positions of the mutations. pET22 is the empty vector. Quantification of the results is given in Table 1.

To determine whether mutations that affect hairpin formation and secretion had an effect on intimin function, we performed adhesion assays with selected mutants on Tir-expressing HeLa cells (Figure 8). In these assays, bacteria expressing Int-Strep, but not IntHA453-Strep or containing the empty vector pET22, bound to cells primed with Tir. The mutants we tested had displayed some effect on either passenger secretion or hairpin formation in immunofluorescence microscopy (Table 1 and Figure 8). Only mutants in the Int-Strep background were tested, as IntHA453-Strep, which does not secrete the C-terminal Tir-binding region to the bacterial surface, is not expected to mediate any adhesion. We also included Int-Strep L437A, which did not have a major effect on either hairpin formation or secretion (Figure 6). Most of the tested mutants bound to Tir-expressing cells at levels comparable to or even higher than the wildtype (Figure 8 and Table 1). Reductions were seen for the two mutants where the extracellular loops were removed (Int-Strep Δloop4 and Δloop5), though these results did not reach statistical significance (*P*<0.15, one-way ANOVA with Dunnett’s multiple comparisons test). However, the Int-Strep Δβ-strand mutant completely failed to mediate adhesion. Adhesion of bacteria expressing the mutants shows that the C-terminal binding domain is correctly folded and presented at the cell surface, though this is reduced for the Δloop4 and Δloop5 mutants, and abolished for the Δβ-strand mutant, which is consistent with the lack of this protein (Figure 6).

**Table 1:** Adhesion of bacteria expressing intimin variants to Tir^+^ HeLa cells

Intimin variant	Mean bacteria per cell
Int-Strep	7.8 ± 3.1
pET22	0*
IntHA-Strep	0*
Int-Strep Δα-helix	8.1 ± 4.0
Int-Strep R288A	11.2 ± 4.5
Int-Strep L437A	21.4 ± 8.2
Int-Strep Δlinker	12.6 ± 5.1
Int-Strep E447A	15.2 ± 6.2
Int-Strep Y448D	10.5 ± 3.9
Int-Strep Δloop4	3.1 ± 2.4
Int-Strep Δloop5	3.5 ± 3.2
Int-Strep Δβ-strand	0*

The number of bacteria adhering to 10 infected cells from one adhesion experiment was counted and the mean number of bacteria per cell is shown with standard deviation. All mutants in the table are in the Int-Strep background. Statistical analysis was done using one-way ANOVA with Dunnett’s multiple comparisons test; an asterisk indicates significant difference from the WT.

## Discussion

The current model for autotransport posits the formation of a hairpin by the linker and the proximal end of the passenger, which then leads to vectorial folding and secretion of the passenger [20]. The hairpin mechanism has been long established for classical autotransporters [21–25], and has more recently been shown to be correct for trimeric (type 5c) [26] and inverse (type 5e) [10] autotransporters. In addition, the hairpin mechanism has been suggested for two-partner secretion (type 5b) systems [27], though there is debate about this issue [28,29]. However, how the hairpin is originally formed is mostly unknown.

In trimeric autotransporters, an ‘ASSA’ region was identified within the linker with reduced helical propensity that was hypothesized to be the initiation site for hairpin formation [30]. Introducing proline residues into this region resulted in a partially stalled secretion intermediate [26]. In type 5a secretion systems, a conserved 14-residue sequence in the linker was identified [31]. Mutations in this region revealed that seven of these residues were crucial for passenger secretion. This contrasts with our observations, where individual mutations in the linker or the β-barrel lumen had no effect on hairpin formation or passenger secretion of the inverse autotransporter intimin. Only once the entire linker was changed to a flexible glycine-serine sequence did we see a reduction in surface exposure. However, even then some protein could be secreted and was functional, as shown by adhesion to Tir-expressing cells. This suggests that when the linker is made flexible, a hairpin can still be formed in a subset of cases leading to passenger secretion. However, the low levels of protein produced in the Int-Strep Δlinker variant indicate that protein where the hairpin is not properly formed is degraded, as is seen for the Δβ-strand variant in the Int-Strep background. Interestingly, in the IntHA453 background the Y448D and Δβ-strand variants were not degraded, suggesting that the double HA tag somehow protects these proteins from being proteolysed.

The role of the β-barrel in autotransporter biogenesis is also not fully established. Apart from being (part of) a secretion pore, there is evidence for extracellular loop 5 of the classical autotransporter Pet forming a scaffold that acts as a template for passenger folding [32]. In EspP, intrabarrel residues mediate the autoproteolysis of the linker to release the passenger into the extracellular medium [33]. A study investigating the role of conserved residues in a type 5a β-barrel found mutations that had an effect on outer membrane integration and passenger secretion [34]. These were mostly in aromatic residues involved in the so called β-signal needed for BAM recognition [35,36], the aromatic girdle at the hydrophobic membrane interface [37], in a structural mortise-tenon motif [38,39], or suspected to be involved in autoproteolysis. In the trimeric autotransporter YadA and inverse autotransporter intimin, exchanging a conserved glycine in a conserved mortise-tenon motif for larger residues impaired passenger secretion, suggesting a narrowing of the secretion pore [7,17]. Recently, the C-terminal β-strand 12 and its final aromatic residue (part of the β-signal) were shown to be necessary for the biogenesis of the inverse autotransporter YeeJ, but replacing β-strand 1 with the sequence of β-strand 3 did not have an effect, showing that the β-barrel can tolerate large changes [40]. This is consistent with our results, as individual point mutations in the intimin β-barrel had hardly any effect. It is likely the several concomitant changes are needed to reduce secretion efficiency, but even large changes such as replacing the linker or loops are well tolerated.

Our main finding is that the β-sheet at the extracellular surface of the intimin β-barrel, formed between the linker and extracellular loops 4 and 5, is crucial for maintaining intimin expression levels. When the residues in the β-strand at the C-terminus of the linker were replaced with glycines, the protein was no longer produced. This could either be due to failure at early steps of biogenesis pathway, e.g. interaction with the BAM before β-barrel closure and outer membrane insertion, or at a later stage due to destabilization of the protein. We prefer the former explanation, as destabilization would probably lead to reduced protein levels or detectable degradation products, not absence entirely. As no protein could be detected in a DegP-negative strain either, this suggests the problem occurs early in the biogenesis pathway and no protein makes it into the outer membrane.

Again, it is surprising that individual mutations in the β-strand (E447A, Y448D) did not have that great an effect on passenger secretion. The Y448D mutation did, however, lead to loss of hairpin formation, but not to the loss of protein in the outer membrane, suggesting that in this case backsliding of the hairpin into the periplasm could be the explanation for why no HA tag was detected at the bacterial surface. In the IntHA453-Strep background, the mutation Y448D disrupts the hydrophobic cluster at the mouth of the β-barrel, and the introduced aspartate may be excluded from the hydrophobic environment, explaining why the backsliding might occur. In Int-Strep, where the D00 domain is able to fold, folding of the first extracellular Ig-like domain could be enough to prevent backsliding, explaining why the Y448D mutation did not have a large effect in the Int-Strep background. However, even in this scenario the hairpin must form at least transiently to allow for passenger secretion, so any exclusion of Y448D and backsliding must be a relatively slow process compared with the folding of D00, which would prevent any backsliding. Partial backsliding could also explain the bimodal distributions seen in flow cytometry for the R434 and Δloop4 variants the IntHA453-Strep background.

It is also interesting to note that deletion of the individual loops 4 or 5 did not have that great an effect. Loop 4 is the larger of the two loops and removing this reduces the amount of protein displayed on the cell surface. However, for both loop deletion mutants, bacteria were still able to bind to Tir-displaying mammalian cells, albeit at reduced levels, showing that the exported protein is correctly folded. In these variants, the other loop is still in place, and this might be enough to allow formation of a β-sheet between the remaining loop and the β-strand at the top of the linker, allowing for some passenger secretion. We expect that deletion of both loops would lead to abolishing secretion and probably protein stability completely, as seen for the Δβ-strand variant.

There is currently no experimental structure of intimin showing the connection between the β-barrel domain and the first extracellular Ig-like domain, D00. However, in the AF model of this region, the connector between the β-strand and the D00 domain is quite long (four extended residues; see Supplementary Figure S12). If this connector is not projected into the extracellular space by the formation of the small β-sheet, the D00 domain might not reach the outside of the β-barrel and be prevented from folding. Why this would lead to protein degradation is unclear, because in the wild-type HA453 variant the D00 cannot fold at the cell surface. The higher stability of the WT IntHA453 variant could be because the β-sheet at the exit of the β-barrel and hairpin are correctly formed. In the absence of the β-sheet the Int-Strep polypeptide might form partly folded structures in the lumen of the (hybrid) β-barrel preventing β-barrel closure and release from the BAM, leading to activation of stress responses (e.g. BepA [41]) that degrade the mis-assembled intimin.

Apart from the extracellular β-sheet, we did not identify interactions within the intimin β-barrel that would lead to the formation of the hairpin. A caveat of our experimental design is that the interactions we targeted are based on the final structure. Therefore, other residues might be involved in transient interactions in the early stages of hairpin formation. However, the robustness of the process, even with large changes such as deleting the periplasmic α-helical turn and making the linker fully flexible, suggests individual residues are not very important.

In conclusion, we found that the intimin β-barrel domain is largely tolerant of changes and most individual residue exchanges do not affect passenger secretion. Only larger changes, such as replacing the linker traversing the β-barrel lumen, had noticeable effects on passenger secretion. Importantly, the β-sheet at the extracellular face of the β-barrel appears to be crucial not only for passenger secretion but protein expression levels overall. This may be because perturbing this region prevents the D00 domain from reaching the outside of the cell to initiate passenger folding and secretion.

## Methods

### Strains and growth conditions

For cloning, *E. coli* TOP10 (Invitrogen) was used. Expression of intimin and its variants was done using *E. coli* BL21ΔF [42], which is equivalent to the BL21(DE3)omp2 strain [43] used in previous studies lacking the abundant porin OmpF for improved outer membrane protein production [7,10,13]. A *degP*-negative version of the BL21ΔF strain (BL21ΔF *degP*) was used to check the effect of DegP [44]. For flow cytometry experiments, BL21Gold(DE3) (Agilent) was employed due to the unavailability of the BL21ΔF strain at the time. However, there is little to no difference in the ability of these strains to produce intimin (Supplementary Figure S14). BL21Gold(DE3) was also used for BN PAGE and protease shaving experiments. For adhesion assays, EPEC E2348/69 Δ*eae* was used [7]. *E. coli* was propagated on lysogeny broth (LB) medium [45] at 37°C (TOP10) or 30°C (BL21ΔF). Autoinduction was performed in ZYP-5052 medium [46] for 20 h at 30°C. Where necessary, media were supplemented with ampicillin at 100 μg/ml. For adhesion assays, bacteria (EPEC, BL21ΔF) were grown in liver medium [7] at 37 or 30°C, respectively.

### Cloning and site-directed mutagenesis

To make use of the convenience of autoinduction, we re-cloned intimin and intimin-HA453 from constructs based on the pASK-IBA2 plasmid [7,10] into the pET22b+ (Novagen), with a PelB signal peptide. This was done using Gibson assembly [47]. Briefly, both insert and vector were amplified by PCR (using Q5 polymerase from New England Biolabs; primer sequences are given in Supplementary Table S1) and the resulting products assembled and transformed into TOP10. Transformants were selected on LB + ampicillin at 100 μg/ml and insert-positive clones were screened for using colony PCR. The correctness of the constructs was verified by Sanger sequencing (Eurofins GmbH or Source Bioscience Genomics). Mutations were introduced by a PCR-based site-directed mutagenesis protocol according to [48]. Briefly, primers with overlaps at their 5’ ends incorporating the mutations were designed to amplify the entire plasmid. The template was removed by DpnI digestion (Fermentas), after which the product was transformed into TOP10 cells and transformants were selected for on LB + ampicillin. Mutations were verified by Sanger sequencing (Eurofins GmbH). All primer sequences are given in Supplementary Table S1.

### Outer membrane isolation

Small-scale outer membrane isolation was performed essentially as described in [49]. Briefly, BL21ΔF strains containing intimin expression plasmids were grown overnight in 50 ml autoinduction medium at 30°C. The following day, an amount of culture equivalent to 40 ml at an optical density at 600 nm (OD_600_) of 1.0 was pelleted and resuspended in 1 ml of lysis buffer (10 mM HEPES pH 7.5, 1 mM MgCl_2_, 1 mM MnCl_2_, 0.1 mg/ml lysozyme, and 10 μg/ml of DNase I). The cells were lysed using a bead beater (Precellys Evolution, with 0.1 mm glass beads). Cell debris were pelleted by a 2-min centrifugation at 15,600 × ***g*** after which membranes were pelleted by centrifuging for 30 min at 20,000 × ***g***. The inner membrane was solubilized with 1% *N*-lauroylsarcosine for 30 min, after which outer membranes were recovered by centrifuging for 30 min at 20,000 × ***g***. The membranes were washed once with 10 mM HEPES pH 7.5, after which the outer membranes were resuspended in 40 μl of 10 mM HEPES pH 7.5 and 10 μl of non-reducing 5 × SDS–PAGE loading dye was added. Samples were stored at −20°C until use.

### Western blots

Outer membrane samples were split in half, and one aliquot was heated for 10 min at 95°C before loading onto an SDS–PAGE gel (Novex gels from Invitrogen). The other half was kept at room temperature before loading. For heat shifts, 4–12% gradient gels were used. The proteins were separated on the gel and then transferred to a nitrocellulose membrane (Whatman Protran) with a Bolt miniblot module (Invitrogen). Once transferred, the membrane was blocked with PBS + 2% skimmed milk powder, either for 1 h at room temperature or overnight at 4°C. The primary antibody (rabbit anti-Strep, Thermo PA5-14454, 1:2000, or rabbit anti-intimin, 1:1000 [7]) was diluted in blocking buffer and incubated with the membrane for 1 h at room temperature. The membrane was then washed three times with PBS-T (PBS + 0.05% Tween20), after which the secondary antibody (goat anti-rabbit CF770, Biotium) was added diluted 1:10,000 in blocking buffer. After 1 h at room temperature, the membrane was washed three times as above and then air dried before imaging with a LI-COR Odyssey CLx imager. For whole cell samples, cells were prepared as in the first steps of outer membrane preparation. After the 2-min centrifugation, a sample was taken for SDS–PAGE and heated for 10 min at 95°C, after which the procedure was the same as above.

### Blue native–PAGE

Outer membrane protein samples for BN–PAGE were prepared as described previously for our denaturing SDS–PAGE experiments with the following minor changes: For intimin expression, *E. coli* BL21Gold (DE3) cells with intimin plasmids were grown o/n in 5 ml LB supplemented with ampicillin at 30°C. The next day, 100 ml autoinduction medium supplemented with ampicillin were inoculated with 2 ml of the LB overnight culture and incubated 20 h at 30°C for Intimin expression. The following day, an amount of culture equivalent to 40 ml at an optical density at 600 nm (OD_600_) of 1.0 was pelleted and processed as described above. After the final wash step with 10 mM HEPES pH 7.5, outer membranes were resuspended in 40 μl resuspension buffer (10 mM HEPES pH 7.5, 10 mM EDTA), supplemented with freshly prepared dodecylmaltoside [0.4% (w/v) final)] and stored at 4°C until BN–PAGE analysis. For BN–PAGE analysis followed the protocol from Zilkenat *et al.* with minor modifications [50]. In short, 18 μl outer membranes in resuspension buffer were mixed with 2 μl BN loading buffer [5% Serva Blue G (Serva), 250 mM aminocaproic acid, 25% glycerol in ddH_2_O] and loaded onto a 4–16% NativePAGE Bis–Tris Mini Protein Gel (Invitrogen). As protein standard, 10 μl NativeMark Unstained Protein Standard (Invitrogen) were used. BN–PAGE was performed at 4°C with BN anode buffer (50 mM Bis–Tris–HCl, pH 7). First, the gel was run at 130 V for ∼1 h using BN cathode buffer A [50 mM Tricine, 15 mM Bis–Tris, 0.02% (w/v) Serva Blue G] until the Coomassie front traversed 1/3 of the gel. Next, BN cathode buffer A was exchanged with a 1:10 dilution of BN cathode buffer B (500 mM Tricine, 150 mM Bis–Tris) in ddH_2_O containing 10% (final) BN cathode buffer A from the cathode chamber and the gel was run at 300 V for ∼1.5 h until the Coomassie front reached the end of the gel. To visualize the protein marker, the part of the gel containing the marker lane was removed and stained separately with Coomassie [0.02% (w/v) Serva Blue G, 5% (w/v) aluminium sulfate hexadecahydrate, 10% (v/v) ethanol, 2% (v/v) ortho-phosphoric acid 85%] overnight. After BN–PAGE, western blot was performed with the part of the gel containing the outer membrane samples to detect Intimin. Therefore, the BN–PAGE gel was equilibrated in SDS–PAGE running buffer [24.8 mM Tris base, 192 mM glycine, and 0.1% (w/v) SDS] for 30 min prior to western blot sandwich assembly. Western blot transfer onto PVDF membranes (activated in EtOH prior to use) was performed in transfer buffer [24.8 mM TRIS base, 192 mM glycine, 0.025% (w/v) SDS, and 10% ethanol] for 3 h at constant 35 V at 4°C. After transfer, the membrane was rinsed 3× in methanol to remove Coomassie from the membrane and rehydrated in dH_2_O for downstream analysis. Membranes were blocked with 2% skimmed milk in PBS for 1 h at room temperature. Primary antibody (rabbit anti-Strep-tag II, abcam ab76950, 1:5000) was diluted in blocking buffer and incubated for 1 h at room temperature. Next, the membrane was washed 3× with PBS-T (PBS + 0.05% Tween20) prior to incubation with the secondary antibody (goat anti-rabbit POX, Thermo 31460, 1:5000) diluted in blocking buffer for 1 h at room temperature. Finally, the membrane was washed 3× with PBS-T followed by one wash step with PBS prior to Luminescence signal detection using Clarity Western ECL Substrate (Bio-Rad).

### Protease shaving assay

For protease shaving experiments, cultures producing intimin variants with an N-terminal (i.e. periplasmic) StrepII were set up in autoinduction medium and grown overnight at 30°C. We used BL21Gold cells for this rather than the BL21ΔF strain for this to be certain that the lack of a major porin would not lead to a compromised outer membrane. The following day, cells were harvested as for outer membrane isolations in duplicate and resuspended in 1 ml 10 mM HEPES pH 7.5 + 1 mM CaCl_2_. To one of the duplicates, proteinase K was added to 50 μg/ml and the cells were incubated 10 min on ice, after which phenylmethysulfonyl fluoride was added to 0.5 mM to both duplicates and the cells were incubated a further 5 min on ice. The bacteria were then washed twice with 10 mM HEPES pH 7.4 and outer membrane isolation was performed as above.

### Immunofluorescence microscopy

For immunofluorescence staining, cultures were auto-induced in 5 ml ZYP-5052 auto-induction medium and grown overnight. About 2 × 10^7^ cells in 1 ml PBS were collected, by measuring OD_600_, on polyethyleneimine-coated 10 mm coverslips in a polystyrene 24-well microtitre plate by centrifugation at 4000 × ***g*** for 5 min. The coverslips were washed with PBS to remove cells that did not stick to the coverslip. The cells were then fixed with 200 μl of 4% paraformaldehyde in PBS for 30 min. After fixing, the cells were washed and blocked with 1% bovine serum albumin (BSA) (from VWR chemicals) in PBS at room temperature for 1 h. The primary antibodies were the rabbit anti-intimin [7] (1:200) and rabbit anti-HA antibody (Santa Cruz Biotechnology, 1:100) in 1% BSA were added and incubated for 1 h at room temperature. The coverslips were washed again three times with PBS-T following which CF488A Goat anti-rabbit antibody (1:200, from Biotium) in 1% BSA was added and incubated in the dark for 2 h at room temperature. The coverslips were then mounted on a glass slide with 10 μl of Biotium’s EverBrite^™^ Mounting Medium with DAPI and sealed around the perimeter with Biotium’s CoverGripTM Coverslip Sealant. The samples were imaged using an UPlanFLN 100×/1.3 oil immersion on an Olympus Fluoview FV1000 Inverted Confocal Microscope. The blue channel (DAPI) was imaged first and then the green channel (CF488A).

### Flow cytometry

For flow cytometry experiments, *E. coli* BL21Gold(DE3) cells with intimin plasmids were grown overnight in 5 ml of autoinduction medium supplemented with ampicillin at 37°C. The cultures were washed with PBS, after which the bacteria were resuspended in 5 ml of PBS and the OD_600_ was measured. 2 × 10^8^ bacteria were pelleted and resuspended in 600 μl of PBS, after which 600 μl of 8% formaldehyde was added and the suspensions incubated for at least 30 min at room temperature. The fixed bacteria were then washed with PBS, after which the cells were resuspended in 1 ml of PBS with 1% BSA and blocked for 1 h at room temperature. The primary antibody [rabbit anti-Strep (Abcam), 1:200 or rabbit anti-HA (Cell Signalling Technology), 1:500] was then added and the cells incubated with rotation overnight at 4°C. The following day, the cells were washed once with PBS, after which the secondary antibody [anti-rabbit CY2 (Abcam), 1:200 in PBS + 1% BSA] was added. The cells were incubated for 1 hour with rotation in the dark at room temperature. The bacteria were washed once with PBS and then resuspended in 300 μl PBS + 1% BSA. Pre-blocked Falcon 5 ml round-bottom tubes were used to inject the bacteria into the flow cytometer FSC-A, SSC-A, and FITC-A signals were recorded in arbitrary units (a.u.) using a BD LSRFortessa^™^ and BD FACSDiva^™^. Detector voltages were set to 489 V (FSC), 225 V (SSC), and 560 V (FITC). A minimum of 10,000 events per sample were collected within a defined gate based on FSC-A versus SSC-A. The results were analysed using Flowjo^™^ (by FlowJo, LLC, a BD company).

### Plasmid stability assays

To assay for potential toxicity of intimin constructs and consequent loss of plasmid, we grew BL21ΔF with intimin constructs or the empty vector pET22b+ in autoinduction medium overnight at 30°C. The following day, the OD_600_ of the cultures was measured and cultures were adjusted to the same OD_600_. From this, bacteria were serially diluted in sterile PBS and 5 μl of dilutions (10^0^–10^−6^) were spotted onto LB or LB + ampicillin 100 μg/ml. Plates were incubated overnight at 30°C and imaged.

### Adhesion assays

Adhesion of intimin-expressing bacteria to HeLa cells was assessed using a prime-challenge assay, essentially as described in [7]. Briefly, HeLa cells were pre-infected with EPEC E2348/69 Δ*eae* at an multiplicity of infection (MOI) of 100 to inject them with Tir. The non-adherent cells were washed off the cells, and any remaining bacteria were killed with gentamicin. After further washes, BL21ΔF containing intimin variants and induced with 0.5 mM isopropylthiogalactoside for 4 h were added to the cells at an MOI of 100 for 1 h. The cells were then washed with PBS until the negative control strain showed no adhesion of bacteria to cells in light microscopy. The samples were then fixed with 4% paraformaldehyde (overnight at 4°C or 1 h at room temperature), washed and stained with Fuchsin for approximately 30 s. The coverslips were washed and air dried and then mounted onto glass slides with the mounting medium Roti^®^ Histokitt. The cells were viewed under an Olympus light microscope at 60× magnification with oil immersion and imaged using Cell B software. For analysis, the number of bacteria adhering to 10 random infected cells in a single experiment was counted manually. ANOVA was performed in GraphPad Prism to determine statistical differences.

### AlphaFold predictions and analysis of intimin mutants

To predict the effects of our mutants onto the intimin protein fold, we generated models using AlphaFold3 (AF, https://alphafoldserver.com/) [14]. As input, we generated intimin sequences of the point mutations I382D, Y448D, and R434A as well as deletion mutants Δα-helix, Δβ-strand, Δloop4, and Δloop5 in the WT background (Figure 1A). In addition, we used the sequence of our IntHA453 construct to predict a model of the stalled variant. All sequences contain the full-length intimin sequence without the N-terminal signal peptide (aa 1–41) to resemble the mature sequence. All sequences are provided in file input.fasta. Besides the amino acid sequences, we included 50 oleate molecules to simulate an artificial membrane plane (details see Results). All AF output files are provided in DATA_VAULT. AF quality scores (pTM, pLDDT), and RMSDs are provided for each model in an Excel file. All these files are available through FigShare [51].

## Supplementary Material

Supplementary Figures S1-S14 and Table S1

## Data Availability

The microscopy images, flow cytometry data and AF models generated in this study are available through FigShare (https://figshare.com/articles/dataset/Intimin_secretion_mutants_microscopy_images/28945613). All other data are included in this paper.

## Abbreviations

AF: AlphaFold3
BAM: β-barrel assembly machinery
BN–PAGE: blue native–polyacrylamide gel electrophoresis
BSA: bovine serum albumin
DAPI: 4′,6-diamidino-2-phenylindole
EHEC: enterohemorrhagic *Escherichia coli*
EPEC: enteropathogenic *Escherichia coli*
HA: haemagglutinin
Ig: immunoglobulin
MOI: multiplicity of infection
OD_600_: optical density at 600 nm
pTM: predicted template modelling score
Tir: translocated intimin receptor
WT: wild-type

## References

[1] Schmidt M.A. (2010) LEEways: tales of EPEC, ATEC and EHEC. Cell. Microbiol. 12, 1544–1552 10.1111/j.1462-5822.2010.01518.x 20716205

[2] Whelan R., McVicker G. and Leo J.C. (2020) Staying out or going in? the interplay between type 3 and type 5 secretion systems in adhesion and invasion of enterobacterial pathogens Int. J. Mol. Sci. 21, 4102 10.3390/ijms21114102 32521829 PMC7312957

[3] Luo Y., Frey E.A., Pfuetzner R.A., Creagh A.L., Knoechel D.G., Haynes C.A. et al. (2000) Crystal structure of enteropathogenic *escherichia coli* intimin-receptor complex. Nature 405, 1073–1077 10.1038/35016618 10890451

[4] Fairman J., Dautin N., Wojtowicz D., Liu W., Noinaj N., Barnard T. et al. (2012) Crystal structures of the outer membrane domain of intimin and invasin from enterohemorrhagic *E. coli* and enteropathogenic *Y. pseudotuberculosis*. Structure 20, 1233–1243 10.1016/j.str.2012.04.011 22658748 PMC3392549

[5] Leo J.C., Oberhettinger P., Chaubey M., Schütz M., Kühner D., Bertsche U. et al. (2015) The intimin periplasmic domain mediates dimerisation and binding to peptidoglycan. Mol. Microbiol. 95, 80–100 10.1111/mmi.12840 25353290

[6] Weikum J., Kulakova A., Tesei G., Yoshimoto S., Jægerum L.V., Schütz M. et al. (2020) The extracellular juncture domains in the intimin passenger adopt a constitutively extended conformation inducing restraints to its sphere of action. Sci. Rep. 10, 21249 10.1038/s41598-020-77706-7 33277518 PMC7718877

[7] Oberhettinger P., Schütz M., Leo J.C., Heinz N., Berger J., Autenrieth I.B. et al. (2012) Intimin and invasin export their C-terminus to the bacterial cell surface using an inverse mechanism compared to classical autotransport. PloS ONE 7, e47069 10.1371/journal.pone.0047069 23056583 PMC3467248

[8] Heinz E., Stubenrauch C.J., Grinter R., Croft N.P., Purcell A.W., Strugnell R.A. et al. (2016) Conserved features in the structure, mechanism, and biogenesis of the inverse autotransporter protein family. Genome Biol. Evol. 8, 1690–1705 10.1093/gbe/evw112 27190006 PMC4943183

[9] Leo J.C., Oberhettinger P., Schütz M. and Linke D. (2015) The inverse autotransporter family: Intimin, invasin and related proteins. Int. J. Med. Microbiol. 305, 276–282 10.1016/j.ijmm.2014.12.011 25596886

[10] Oberhettinger P., Leo J.C., Linke D., Autenrieth I.B. and Schütz M.S. (2015) The inverse autotransporter intimin exports its passenger domain via a hairpin intermediate. J. Biol. Chem. 290, 1837–1849 10.1074/jbc.M114.604769 25488660 PMC4340425

[11] Doyle M.T., Jimah J.R., Dowdy T., Ohlemacher S.I., Larion M., Hinshaw J.E. et al. (2022) Cryo-EM structures reveal multiple stages of bacterial outer membrane protein folding. Cell 185, 1143.e13–1156.e13 10.1016/j.cell.2022.02.016 35294859 PMC8985213

[12] Doyle M.T. and Bernstein H.D. (2021) BamA forms a translocation channel for polypeptide export across the bacterial outer membrane. Mol. Cell. 81, 2000.e3–2012.e3 10.1016/j.molcel.2021.02.023 33705710 PMC8106670

[13] Leo J.C., Oberhettinger P., Yoshimoto S., Udatha D.B., Morth J.P., Schütz M. et al. (2016) Secretion of the intimin passenger domain is driven by protein folding. J. Biol. Chem. 291, 20096–20112 10.1074/jbc.M116.731497 27466361 PMC5025694

[14] Abramson J., Adler J., Dunger J., Evans R., Green T., Pritzel A. et al. (2024) Accurate structure prediction of biomolecular interactions with AlphaFold 3. Nature 630, 493–500 10.1038/s41586-024-07487-w 38718835 PMC11168924

[15] Masters M.R., Mahmoud A.H. and Lill M.A. (2025) Investigating whether deep learning models for co-folding learn the physics of protein-ligand interactions. Nat Commun. 16, 8854–8912 10.1038/s41467-025-63947-5 41053181 PMC12501370

[16] Noinaj N., Kuszak A.J. and Buchanan S.K. (2015) Heat modifiability of outer membrane proteins from gram-negative bacteria. Methods Mol. Biol. 1329, 51 10.1007/978-1-4939-2871-2_4 26427675 PMC4880013

[17] Grosskinsky U., Schütz M., Fritz M., Schmid Y., Lamparter M.C., Szczesny P. et al. (2007) A conserved glycine residue of trimeric autotransporter domains plays a key role in *yersinia* adhesin A autotransport. J. Bacteriol. 189, 9011–9019 10.1128/JB.00985-07 17921300 PMC2168626

[18] Ruiz-Perez F., Henderson I.R., Leyton D.L., Rossiter A.E., Zhang Y. and Nataro J.P. (2009) Roles of periplasmic chaperone proteins in the biogenesis of serine protease autotransporters of *enterobacteriaceae*. J. Bacteriol. 191, 6571–6583 10.1128/JB.00754-09 19734313 PMC2795308

[19] Purdy G.E., Fisher C.R. and Payne S.M. (2007) IcsA surface presentation in *shigella flexneri* requires the periplasmic chaperones DegP, skp, and SurA. J. Bacteriol. 189, 5566–5573 10.1128/JB.00483-07 17526712 PMC1951818

[20] Bernstein H.D. (2015) Looks can be deceiving: recent insights into the mechanism of protein secretion by the autotransporter pathway. Mol. Microbiol. 97, 205–215 10.1111/mmi.13031 25881492 PMC6376861

[21] Junker M., Besingi R.N. and Clark P.L. (2009) Vectorial transport and folding of an autotransporter virulence protein during outer membrane secretion. Mol. Microbiol. 71, 1323–1332 10.1111/j.1365-2958.2009.06607.x 19170888

[22] Oliver D.C., Huang G., Nodel E., Pleasance S. and Fernandez R.C. (2003) A conserved region within the *bordetella pertussis* autotransporter BrkA is necessary for folding of its passenger domain. Mol. Microbiol. 47, 1367–1383 10.1046/j.1365-2958.2003.03377.x 12603741

[23] Renn J., Junker M., Besingi R., Braselmann E. and Clark P. (2012) ATP-independent control of autotransporter virulence protein transport via the folding properties of the secreted protein. Chem. Biol. 19, 287–296 10.1016/j.chembiol.2011.11.009 22209629 PMC3288764

[24] Peterson J.H., Tian P., Ieva R., Dautin N. and Bernstein H.D. (2010) Secretion of a bacterial virulence factor is driven by the folding of a C-terminal segment. Proc. Natl. Acad. Sci. U.S.A. 107, 17739–17744 10.1073/pnas.1009491107 20876094 PMC2955144

[25] Leyton D.L., Sevastsyanovich Y.R., Browning D.F., Rossiter A.E., Wells T.J., Fitzpatrick R.E. et al. (2011) Size and conformation limits to secretion of disulfide-bonded loops in autotransporter proteins. J. Biol. Chem. 286, 42283–42291 10.1074/jbc.M111.306118 22006918 PMC3234927

[26] Chauhan N., Hatlem D., Orwick‐Rydmark M., Schneider K., Floetenmeyer M., van Rossum B. et al. (2019) Insights into the autotransport process of a trimeric autotransporter, *yersinia* adhesin A (YadA). Mol. Microbiol. 111, 844–862 10.1111/mmi.14195 30600549

[27] Mazar J. and Cotter P.A. (2006) Topology and maturation of filamentous haemagglutinin suggest a new model for two‐partner secretion. Mol. Microbiol. 62, 641–654 10.1111/j.1365-2958.2006.05392.x 16999837

[28] Jacob-Dubuisson F., Guérin J., Baelen S. and Clantin B. (2013) Two-partner secretion: as simple as it sounds? Res. Microbiol. 164, 583–595 10.1016/j.resmic.2013.03.009 23542425

[29] Guérin J., Bigot S., Schneider R., Buchanan S.K. and Jacob-Dubuisson F. (2017) Two-partner secretion: combining efficiency and simplicity in the secretion of large proteins for bacteria-host and bacteria-bacteria interactions. Front Cell Infect. Microbiol. 7, 148 10.3389/fcimb.2017.00148 28536673 PMC5422565

[30] Shahid S.A., Bardiaux B., Franks W.T., Krabben L., Habeck M., Van Rossum B. et al. (2012) Membrane-protein structure determination by solid-state NMR spectroscopy of microcrystals. Nat. Methods 9, 1212–1217 10.1038/nmeth.2248 23142870

[31] Kostakioti M. and Stathopoulos C. (2006) Role of the α-helical linker of the C-terminal translocator in the biogenesis of the serine protease subfamily of autotransporters. Infect. Immun. 74, 4961–4969 10.1128/IAI.00103-06 16926387 PMC1594850

[32] Yuan X., Johnson M.D., Zhang J., Lo A.W., Schembri M.A., Wijeyewickrema L.C. et al. (2018) Molecular basis for the folding of β-helical autotransporter passenger domains. Nat. Commun. 9, 1395 10.1038/s41467-018-03593-2 29643377 PMC5895577

[33] Anderson D.E., Dautin N., Barnard T.J. and Bernstein H.D. (2007) Cleavage of a bacterial autotransporter by an evolutionarily convergent autocatalytic mechanism. EMBO J. 26, 1942–1952 10.1038/sj.emboj.7601638 17347646 PMC1847664

[34] Yen Y.T., Tsang C., Cameron T.A., Ankrah D.O., Rodou A. and Stathopoulos C. (2010) Importance of conserved residues of the serine protease autotransporter β-domain in passenger domain processing and β-barrel assembly. Infect. Immun. 78, 3516–28 10.1128/IAI.00390-10 20515934 PMC2916275

[35] Paramasivam N., Habeck M. and Linke D. (2012) Is the C-terminal insertional signal in gram-negative bacterial outer membrane proteins species-specific or not? BMC Genomics 13, 510 10.1186/1471-2164-13-510 23013516 PMC3582582

[36] Robert V., Volokhina E.B., Senf F., Bos M.P., Gelder P. and Tommassen J. (2006) Assembly factor Omp85 recognizes its outer membrane protein substrates by a species-specific C-terminal motif. PLoS Biol. 4, 1984–e377 10.1371/journal.pbio.0040377 PMC1634882 17090219

[37] Hermansen S., Linke D. and Leo J.C. (2022) Transmembrane β-barrel proteins of bacteria: from structure to function. Adv. Protein Chem. Struct. Biol. 128, 113–161 10.1016/bs.apcsb.2021.07.002 35034717

[38] Leyton D.L., Johnson M.D., Thapa R., Huysmans G.H., Dunstan R.A., Celik N. et al. (2014) A mortise–tenon joint in the transmembrane domain modulates autotransporter assembly into bacterial outer membranes. Nat. Commun. 5, 4239 10.1038/ncomms5239 24967730

[39] Michalik M., Orwick-Rydmark M., Habeck M., Alva V., Arnold T. and Linke D. (2017) An evolutionarily conserved glycine-tyrosine motif forms a folding core in outer membrane proteins. PloS ONE 12, e0182016 10.1371/journal.pone.0182016 28771529 PMC5542473

[40] Schreiber S., Zaayenga A. and Jose J. (2024) The assembly of the inverse autotransporter protein YeeJ is driven by its C-terminal β-strand. J. Mol. Biol. 436, 168749 10.1016/j.jmb.2024.168749 39173735

[41] Narita S., Masui C., Suzuki T., Dohmae N. and Akiyama Y. (2013) Protease homolog BepA (YfgC) promotes assembly and degradation of β-barrel membrane proteins in *Escherichia coli*. Proc. Natl. Acad. Sci. U.S.A. 110, E3612–E3621 10.1073/pnas.1312012110 24003122 PMC3780861

[42] Meuskens I., Michalik M., Chauhan N., Linke D. and Leo J.C. (2017) A new strain collection for improved expression of outer membrane proteins. Front Cell Infect. Microbiol. 7, 464 10.3389/fcimb.2017.00464 29164072 PMC5681912

[43] Prilipov A., Phale P.S., Van Gelder P., Rosenbusch J.P. and Koebnik R. (1998) Coupling site-directed mutagenesis with high-level expression: Large scale production of mutant porins from *E. coli*. FEMS Microbiol. Lett. 163, 65–72 10.1111/j.1574-6968.1998.tb13027.x 9631547

[44] Ryoo D., Rydmark M.O., Pang Y.T., Lundquist K.P., Linke D. and Gumbart J.C. (2020) BamA is required for autotransporter secretion. Biochim. Biophys. Acta Gen. Subj. 1864, 129581 10.1016/j.bbagen.2020.129581 32114025 PMC7196024

[45] Bertani G. (1951) Studies on lysogenesis. I. the mode of phage liberation by lysogenic *escherichia coli*. J. Bacteriol. 62, 293–300 10.1128/jb.62.3.293-300.1951 14888646 PMC386127

[46] Studier F.W. (2005) Protein production by auto-induction in high-density shaking cultures. Protein Expr. Purif. 41, 207–234 10.1016/j.pep.2005.01.016 15915565

[47] Gibson D.G., Chuang R., Hutchison C.A., Venter J.C., Smith H.O. and Young L. (2009) Enzymatic assembly of DNA molecules up to several hundred kilobases. Nat. Methods 6, 343–345 10.1038/nmeth.1318 19363495

[48] Liu H. and Naismith J.H. (2008) An efficient one-step site-directed deletion, insertion, single and multiple-site plasmid mutagenesis protocol. BMC Biotechnol. 8, 91 10.1186/1472-6750-8-91 19055817 PMC2629768

[49] Leo J.C., Oberhettinger P. and Linke D. (2015) Assessing the outer membrane insertion and folding of multimeric transmembrane beta-barrel proteins. Methods Mol. Biol. 1329, 157–167 10.1007/978-1-4939-2871-2_12 26427683

[50] Zilkenat S., Kim E., Dietsche T., Monjarás Feria J.V., Torres-Vargas C.E., Mebrhatu M.T. et al. (2024) Blue native PAGE analysis of bacterial secretion complexes. Methods Mol. Biol. 2715 331–362 10.1007/978-1-0716-3445-5_22 37930539

[51] Sarma Kandanur S.P. and Leo J.C. (2025) Intimin secretion mutants microscopy images.FigShare. Dataset. https://doi.org/10.6084/m9.figshare.28945613.v3

