# Supplementary material for: Mutations affecting the β-sheet at the extracellular opening of the intimin β-barrel domain lead to reduced protein levels and inefficient passenger secretion
Source: Biosci Rep. 2026 Feb 6;46(2):BSR20253581. doi: 10.1042/BSR20253581 (PMC13078405; doi:10.1042/BSR20253581)
Supplement: Supplementary Figures S1-S14 and Table S1 [file BSR-2025-3581_supp.pdf]

Supplementary Material for Sarma et al.

Supplementary Information

AlphaFold analysis of intimin mutants

In order to assess the effects of our mutations on the final folded state of intimin, we generated models using AlphaFold3 (AF) [1]. As AF does not consider the biogenesis of a protein, we focused on the mutations in the WT background which have completed their biological assembly and only modeled the IntHA453 stalled variant without any mutations as reference. Initial predictions were generated using only the amino acid sequence of intimin. However, we found that AF tends to place 1-3 helices (aa 151-202) with low predicted Local Distance Difference Test (pLDDT) and high predicted aligned error (PAE) confidence scores next to the β-barrel domain (not shown). pLDDT provide confidence estimates of the AF predictions on per-atom basis while PAE serves as an error estimate in the relative positioning of e.g. two domains to each other. Hence, both low pLDDT and high PAE are indicators of locally not reliable AF predictions. The presence of those helices in close contact with residues of the β-barrel impacted the pLDDT scores of the β-barrel. As a transmembrane (TM) helix between the intimin LysM and β-barrel domain was not reported previously, we chose to predict its transmembrane helix and disorder properties using DeepTMHMM [2] and IUPred2A [3,4], respectively. For this, we used the intimin sequence with the signal peptide which served as an internal control. DeepTMHMM and IUPred2A indicate no presence of a TM helix or an ordered region in this region (Supplementary Figure 4A, B). Together with the confidence scores, no predictions of a TM helix, and manual inspection, we concluded that these helices are due to AF hallucinations as reported previously [1]. As we used pLDDT scores to access the effect of the mutations onto different parts of intimin, we chose to include 50 oleate molecules in our AF predictions to form an artificial membrane plane (as suggested in the AF guide, https://alphafoldserver.com/guides#section-2:-alphafold-server:-your-gateway-to-alphafold-3). This resulted in the placement of several oleate molecules in between the β-barrel and the hallucinated helices, thereby preventing the influences of the hallucinated helices on residues in the β-barrel (supplementary Figure 4C-M, data file of all predictions available in FigShare).

As each AF run returns five different models, we choose to analyze all five models of each prediction. To compare overall fold quality, we chose the AF predicted template modeling score (pTM) instead of the interface predicted template modeling score (ipTM) as the ipTM contains information about the clearly artificial oleate membrane plane. Almost all AF predictions yielded pTM scores above 0.5 indicating high confidence fold predictions (Excel file of predictions available in FigShare)[1]. The highest pTM scores of 0.562 ± 0.007 was observed for the WT protein and all other mutants tested reached similar high scores. The only two mutants with pTM scores below 0.5 are the IntHA453 variant with 0.404 ± 0.008 and the Int Δloop4 mutant 0.482 ± 0.007 indicating overall problems of AF to achieve high confidence predictions. As those two intimin variants also displayed one of the most pronounced phenotypes in comparison to the WT protein, we were not surprised.

Furthermore, we aligned all models belonging to one mutant in PyMol (Schroedinger) and use the resulting RMSD between the structures as an indicator for overall similarity in domain arrangement predictions of that mutant. Non surprising, the only prediction which did not return very similar models with RMSDs below 4.5 Å was the IntHA453 variant with an RMSD of 22.325 ±

1.560 Å. Intermodel variations in domain organization was observed within the periplasmic LysM domains and the remaining unstructured periplasmic sequences including the hallucinated helices (Supplementary Figure 4C-M). In addition to this flexibility, presence of the HA-tag yielded to a loss in coherence of the extracellular domains and the β-barrel domain but were unable to capture the stalled hairpin as suggested from previous research [5,6]. This is not surprising, given the scope of AF to only model the final structure of a protein and not its biogenesis.

As AF predictions are usually not suitable to resolve the effects of mutations onto the fold of a protein due to its inherent properties and due to its lack of physical and chemical knowledge as a result of its training as reported recently [7], we opted to focus on the pLDDT scores for evaluation the predicted effects of our mutations on the intimin folding. To resolve local disturbances of the mutants, we chose to average and compare the per-atom pLDDT confidence scores of several key regions across all atoms belonging to this region (main chain and side chain heavy atoms). Key regions were the periplasmic LysM domain (aa 20-74) which is supposed to be not affected by any mutations and serves as internal control, the first β-strand of the β-barrel domain (barrel β1, aa 169-180) and the last β-strand of the β-barrel domain (barrel β12, aa 355-370) as proxies for the stability of the intimin β-barrel as we observed here the largest in the β-barrel, regions containing our mutations: loop4 (aa 268-286, Figure 1), loop5 (aa 313-328, Figure 1), the periplasmatic α-helix (aa 371-389, Figure 1), the linker in the lumen of the β-barrel (aa 393-403, Figure 1), the extracellular β-strand (aa 405-408, Figure 1), the extracellular connector between the β-strand and the D00 domain (aa 409-412, Supplementary Figure 8), and finally the extracellular D00 domain (aa 413-510). The connector and the D00 domain were selected to analyze the effects of our mutations onto the folding of the extracellular parts of intimin. Note, that the amino acid numbers provided correspond to the sequences without the N-terminal signal sequence (aa 1-41 of uniport ID P19809). We extracted all average pLDDT scores for all five models of each analyzed construct for comparison (Supplementary Figure pLDDT 5, Excel file available in FigShare). In order to summarize our AF predictions, we evaluated the pLDDT changes induced by our mutations on each region by calculating the difference (ΔpLDDT) of each model's pLDDT of one region to the average pLDDT of WT prediction of the same region (Figure average ΔpLDDT). A decrease of the average ΔpLDDT indicates a decrease in the local AF folding confidence and hence might be read as a predicted destabilization of the local fold. Of note, stabilizing effects are rarely observed and less pronounced than destabilizing effects.

The presence of the HA-tag clearly influences the predictions of intimin across its whole structure and the most pronounced ΔpLDDT changes are observed next to the HA-tag in the connector which is highly destabilized with an ΔpLDDT = -18.140. In addition, the destabilizing effect is translated throughout the whole structure from the extracellular D00 domain to the periplasmic α-helix. This is in line with the experimental observations of stalled intimin biogenesis in this variant [6]. The effects of our mutants usually do not translate across the whole intimin structure and are more localized. Also, the effects of single amino acid substitutions are less pronounced if present at all. E.g. the Δβ-strand mutation destabilizes the β-strand itself (ΔpLDDT = -8.984) and the directly adjacent connector (ΔpLDDT = -7.882), loop4 (ΔpLDDT = -6.130), and loop5 (ΔpLDDT = -3.134) but fails to induce changes within the directly adjacent linker whereas the Y448D mutation within the β-strand only leads to minor destabilizations of the β-strand (ΔpLDDT = -2.198), loop4 (ΔpLDDT = -1.230), and loop5 (ΔpLDDT = -1.606). This again, is in line with the severity of the experimental phenotypes.

It becomes also obvious, that our mutation replacing parts of the molecule with G or G/S stretches (Δβ-strand, Δloop4, Δloop5, Δlinker, Δα-helix) have pronounced local effects with the

largest pLDDT change observed in chase of the Δα-helix mutation of ΔpLDDT = -39.108. However, this large local impact does not translate into the extracellular site in a negative manner but seems to slightly stabilize loop4 and the D00 domain (ΔpLDDT = 0.708 and ΔpLDDT = 1.960, respectively). Its destabilizing effect of the regions in direct contact with the α-helix, i.e. the β12-strand (ΔpLDDT = -4.600) and the β1-strand (ΔpLDDT = -2.778) of the β-barrel and C-terminal the linker (ΔpLDDT = -1.998) in the lumen of the β-barrel, are surprisingly less pronounced. Interestingly, mutations (Δβ-strand, Y448D, Δloop4, Δlinker, R434A) which affect the hairpin biogenesis in our FACS data, that is non-fluorescent cells in the IntHA453 background including the bimodal populations, seem to affect eighter largely the linker (Δlinker, R434A), or the intricate network of loops and secondary structure elements at the extracellular site of the β-barrel (Δβ-strand, Y448D, Δloop4). Why the Δloop5 mutation does not affect the hairpin formation is maybe the results of a slight stabilization of the D00 domain and its lack of an effect on the connector when compared to the Y448D mutation which has an overall smaller effect on the ΔpLDDT values. Structurally, loop5 seems to be less involved in the stabilization of the extracellular β-strand and connector (Figure 1 and Supplementary Figure 8). In line with that, Δloop4 has stronger negative effects onto of the extracellular β-strand (ΔpLDDT = -23.114) and connector (ΔpLDDT = -22.520) than on itself (ΔpLDDT = -14.454). Mutants Δloop4 and IntHA453, which affect translocation of the extracellular domains, the final step of intimin biogenesis, in the WT background have the largest destabilizing effect on the connector (ΔpLDDT = -18.140 and ΔpLDDT = -22.520, respectively) which seems to more crucial than D00 folding (ΔpLDDT = -2.502 and ΔpLDDT = -0.544, respectively). Similarly, the Δβ-strand mutation is also predicted to destabilize the connector ΔpLDDT = -7.882). However, we could not detect protein in our Western Blot analysis (Figure 6B) indicating severe problems in the biogenesis of the β-barrel. Interestingly, the Δβ-strand mutation was the only mutation which predicted an increased confidence of both β-barrel β-strands analyzed (β1 ΔpLDDT = 1.960, β12 ΔpLDDT = 0.750) but how that correlates with the observed defects in β-barrel biogenesis remains enigmatic.

Table 1. Primers used in this study.

Primers	Sequences (5'→3')	Comment
pET22rec Fwd<br>pET22rec Rev	TGAGATCCGGCTGCTAACAAAG<br>GGCCATCGCCGGCTGG	For amplifying pET22 for cloning
Strep-PelB Rev	GAACTGCGGGTGGCTCCACGCGGCCATCGCCGGCTGG	For amplifying pET22 for cloning and inserting a StrepII tag after the PelB signal sequence
PelB-Int Fwd<br>pET22-Strep Rev	GCCCAGCCGGCGATGGCCAATGGTGAAAATTATTTTAAATTGGGTTC<br>GTTAGCAGCCGGATCTCATTATTTTTCGAACTGCGGGTGG	For amplifying intimin (with C-terminal StrepII tag) for cloning
Strep-Int Fwd<br>pET22-Int Rev	TGGAGCCACCCGCAGTTCGAAAAAAATGGTGAAAATTATTTTAAATTGGGTTC<br>GTTAGCAGCCGGATCTCA TTA TTT TAC ACA AGT GGC ATA AGC ATT AG	For amplifying intimin (with N-terminal StrepII tag) for cloning
IntD298A Fwd<br>IntD298A Rev	AATACTGGCGAGCTTATTTCAAAAGTAGTGTTAACGGCTA<br>AGCTCGCCAGTATTCGCCACCAATACCTAAACGG	Mutagenesis primers to introduce the D298A mutation
IntI382D Fwd<br>IntI382D Rev	AACTATACTCCGGATCCTCTGGTGACGATGGGG<br>ATCCGGAGTATAGTTTACACCAACGGTCGCCGC	Mutagenesis primers to

		introduce the I382D mutation
IntP421A Fwd<br>IntP421A Rev	CAGCAAATTGAGGCTCAATATGTTAACGAGTTAAGAACATTAT<br>AGCCTCAATTTGCTGGGACCACGGTTTATCAAACTGAT	Mutagenesis primers to introduce the P421A mutation
IntL430A Fwd<br>IntL430A Rev	GAGTTAAGAACAGCTTCAGGCAGCCGTTACGATCT<br>AGCTGTTCTTAACTCGTTAACATATTGTGGCTCAATTTGC	Mutagenesis primers to introduce the L430A mutation
IntΔα-helix Fwd<br>IntΔα-helix Rev	TTTTCTGGTTCTGGTTCAGGCAGCCGTTACGATCT<br>ACCAGAACCAGAAAACTGATAACGGAACTGCATTGAGT	Mutagenesis primers to replace residues 412-430 with SGSG
IntF276A Fwd<br>IntF276A Rev	GCTATAACGTCGCCATTGATCAGGATTTTTCTGGTGATAAT<br>GGCGACGTTATAGCCCAACATATTTTCAGGAAGGAAAAAA	Mutagenesis primers to introduce the F276A mutation
IntR288A Fwd<br>IntR288A Rev	GGTGATAATACCGCGTTAGGTATTGGTGGCGAATACTG<br>CGC GGT ATT ATC ACC AGA AAA ATC CTG ATC AAT GAA GAC G	Mutagenesis primers to introduce the R288A mutation
IntR434A Fwd<br>IntR434A Rev	TCAGGCAGCGCTTACGATCTGGTTCAGCGTAATAA<br>AGCGCTGCCTGATAATGTTCTTAACTCGTTAACATATTGT	Mutagenesis primers to introduce the R434A mutation
IntL437A Fwd<br>IntL437A Rev	GCCGTTACGATGCTGTTCAGCGTAATAACAATATTATTCTG<br>AGCATCGTAACGGCTGCCTGATAATGTTCTTAACTCGTT	Mutagenesis primers to introduce the L437A mutation
IntQ439A Fwd<br>IntQ439A Rev	ACGATCTGGTTGCTCGTAATAACAATATTATTCTGGAGTAC<br>AGCAACCAGATCGTAACGGCTGCCTGATAATGTTCTT	Mutagenesis primers to introduce the Q439A mutation
IntΔlinker Fwd<br>IntΔlinker Rev	AGTGGGTCTGGCTCCGGTAGCATTCTGGAGTACAAAAAGCAGGATA<br>GCCAGACCCACTACCGCTGCCACGGCTGCCTGATAATGTTCTT	Mutagenesis primers to replace residues 435-444 with GSGSGSGSGS
IntE447A Fwd<br>IntE447A Rev	ACAATATTATTCTGGCGTACAAAAAGCAGGATATTCTTTCTC<br>CGCCAGAATAATATTGTTATTACGCTGAACCAGATCGTAA	Mutagenesis primers to introduce the E447A mutation
IntY448D Fwd<br>IntY448D Rev	ATTATTCTGGAGGACAAAAAGCAGGATATTCTTTCTCTGA<br>GTCCTCCAGAATAATATTGTTATTACGCTGAACCAGATC	Mutagenesis primers to introduce the Y448D mutation
IntΔloop4 Fwd<br>IntΔloop4 Rev	GGTGGGGGTGGGAATGGCTTCGATATCCGTTTTAAT<br>CCCACCCCCACCGAAATAGCCGTTAACACTACT TTT G	Mutagenesis primers to replace residues 309-327 with GGGG
IntΔloop5 Fwd<br>IntΔloop5 Rev	GGTGGACCTGGTGCGGCGACCGTTGGTGTA<br>ACCAGGTCCACCTCCACCATACTGCTCATACATCA	Mutagenesis primers to replace residues 354-369 with GGGG
IntΔβ-strand Fwd<br>IntΔβ-strand Rev	GGTGGGGGTGGGAAGCAGGATATTCTTTCTCTGAATATT<br>CCCACCCCCACCAATAATATTGTTATTACGCTGAACCAG	Mutagenesis primers to replace residues 446-449 with GGGG

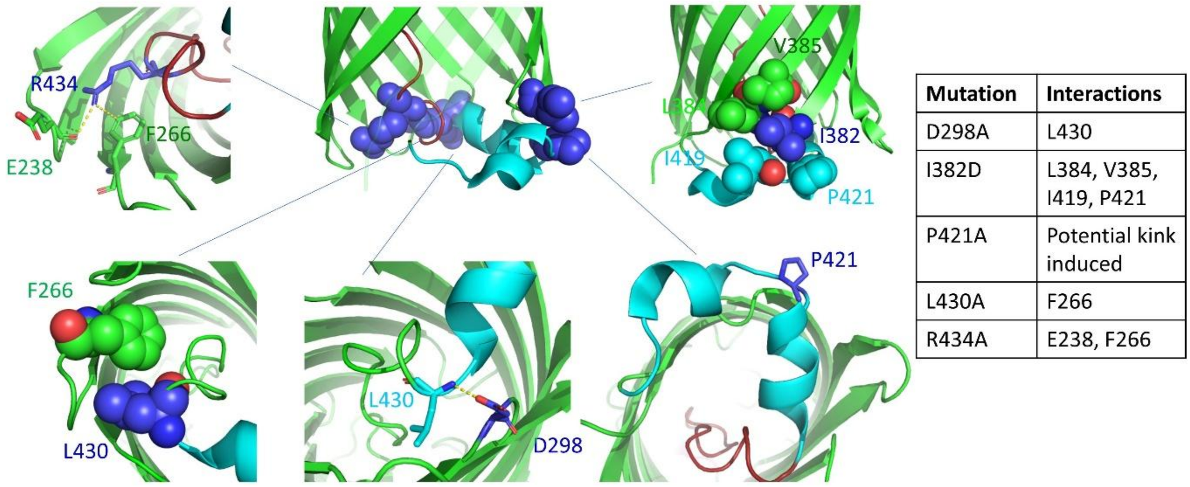

Mutation	Interactions
D298A	L430
I382D	L384, V385, I419, P421
P421A	Potential kink induced
L430A	F266
R434A	E238, F266

Supplementary Figure 1. Residues and interactions targeted at the periplasmic side of the intimin β-barrel domain. Mutated residues are shown in blue, in either space-filling or stick representation; interacting residues are coloured according to the colour scheme in Figure 1 in the main paper, and hydrogen or ionic bonds in dashed yellow lines. D298 interacts with the amide nitrogen of L430, potentially stabilising the N-terminus of the linker. I382 forms the core a hydrophobic pocket involving L384, V385, I419 and P421. P421 is situated in a turn between two short α-helices and may be important in introducing a kink at this position. L430 has a hydrophobic interaction with F266, which may be important in positioning the N-terminus of the linker. R434 in the linker has an ionic interaction with E238 in a periplasmic turn and a cation-pi interaction with F266 in the lumen wall. These interactions are summarised in the table on the right. The Δα-helix mutation replaces the entire helical turn (in cyan in the middle) with a glycine-serine stretch of the same length. The figures were prepared using PyMOL (Schroedinger).

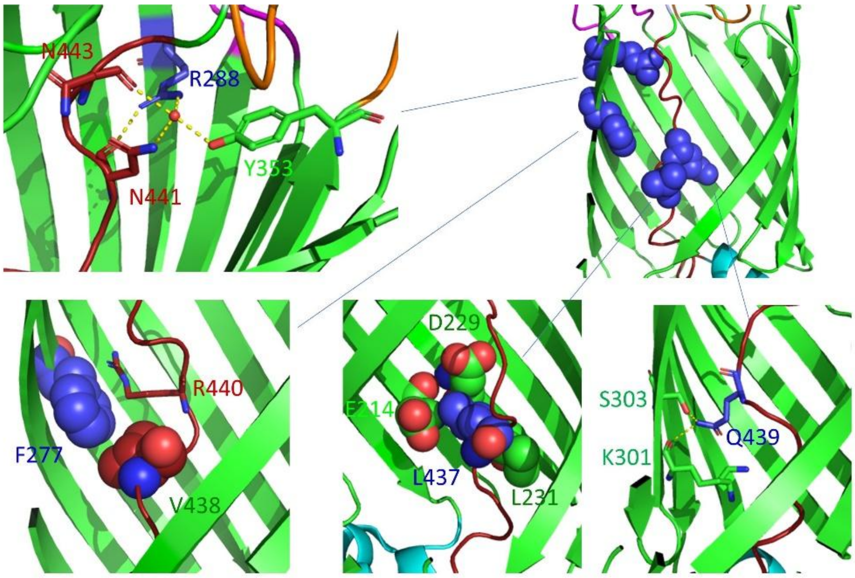

Mutation	Interactions
D277A	V438, R440
R288A	Y353, N441, N443
L437	E214, D229, L231
LQ439	K301, S303

Supplementary Figure 2. Residues and interactions targeted in the linker region of intimin. Mutated residues are shown in blue, in either space-filling or stick representation; interacting residues are shown according to the colour scheme in Figure 1 in the main paper, and hydrogen or ionic bonds in dashed yellow lines. F277, located on the lumen wall, has a hydrophobic interaction with V438 as well as cation-pi interaction with R440. R288, also on the lumen wall, interacts through with several residues including N441 and N443 in linker either directly or through a water bridge. L437 in the linker packs against L231, E214 and D229, located in the lumen wall. Q439 in the linker forms hydrogen bonds with K301 and S303 on the lumen wall. These interactions are summarised in the table on the right. In addition, the entire linker (434-444) was replaced by a GS sequence of the same length. The figures were prepared in PyMOL (Schroedinger).

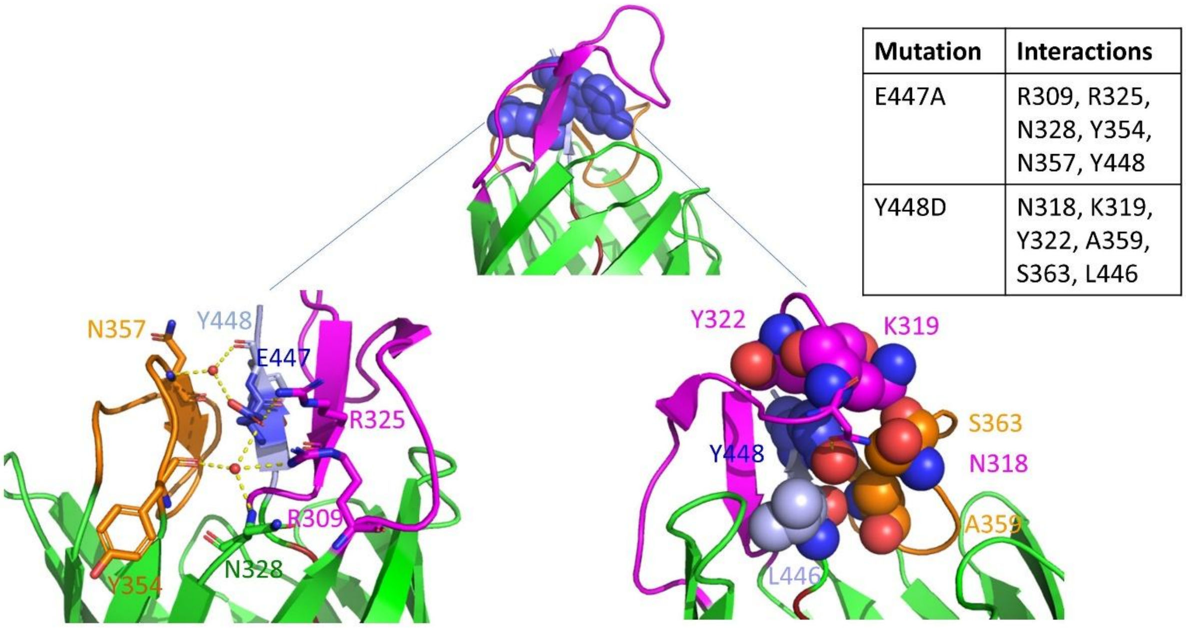

Mutation	Interactions
E447A	R309, R325, N328, Y354, N357, Y448
Y448D	N318, K319, Y322, A359, S363, L446

Supplementary Figure 3. Residues and interactions targeted at the extracellular region of intimin. Mutated residues are shown in blue, in either space-filling or stick representation; interacting residues are shown according to the colour scheme in Figure 1 in the main paper, and hydrogen or ionic bonds in dashed yellow lines. E447, in the β-strand at the C-terminal end of the linker, is at the centre of an interaction network between several residues in the neighbouring loops. Y448 is at the core of a hydrophobic cluster involving residues from loop 4 (N318, K319, Y322) loop 5 (A359, S363) and the linker (L446). These interactions are summarised in the table on the right. In addition, both loops 4 and 5 were replaced by a four-glycine stretch (Δloop4 and Δloop5, respectively), as was the β-strand itself (Δβ-strand). The figures were prepared in PyMOL (Schroedinger).

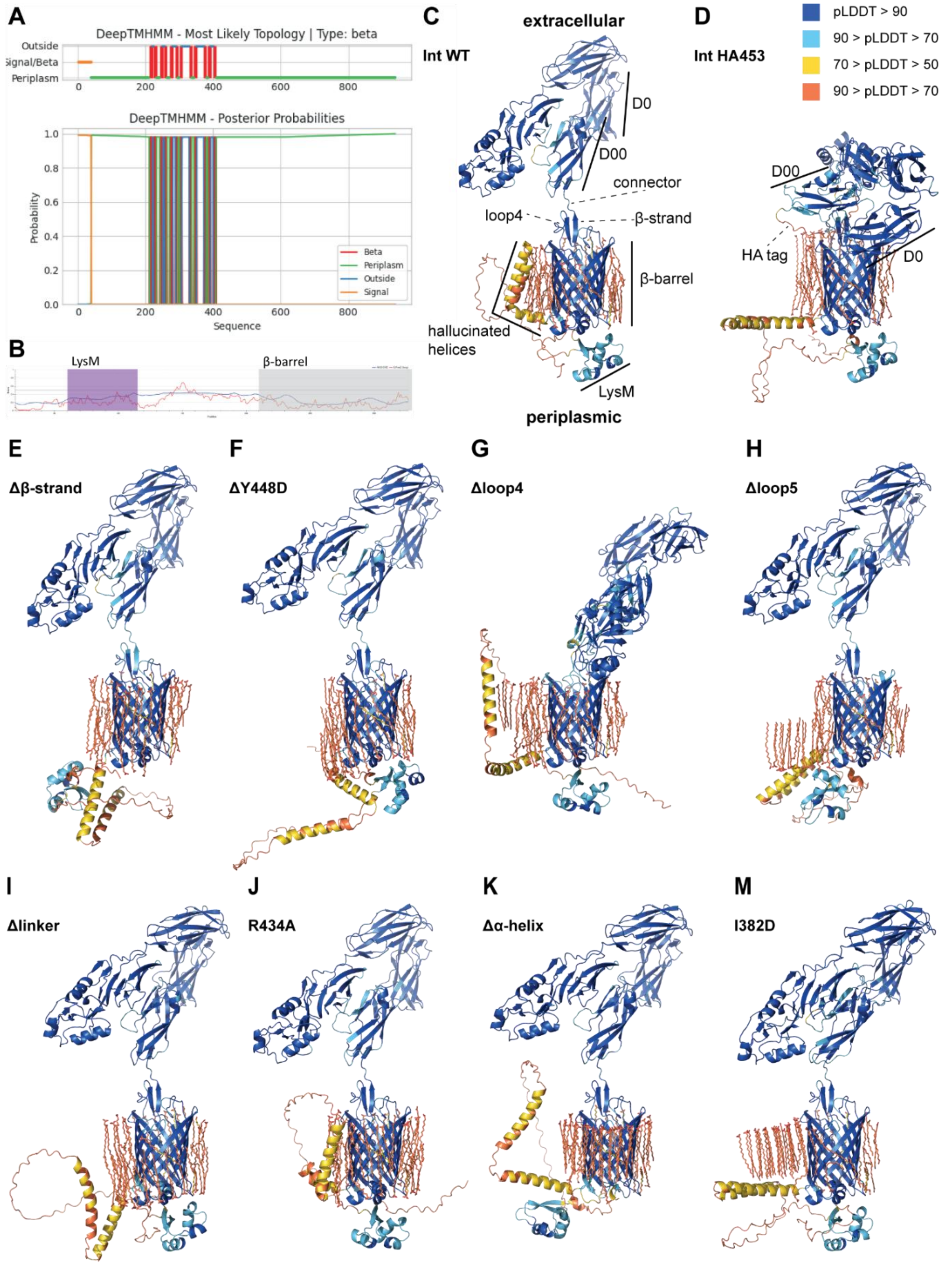

Supplementary Figure 4. AF predictions of FL intimin mutations. A) + B) The region between the LysM and β-barrel does not contain a transmembrane helix and is disordered. DeepTMHMM [2] (A) does not predict the presence of transmembrane helices between the N-terminal export signal and the β-barrel domain (aa 210-411). B) IUPred2A [3,4] (B) predicts a high degree of disorder C-terminal of the LysM domain (aa 61-115, purple box) and N-terminal of the β-barrel domain (grey box). C) AF model of Intimin WT. Note the presence of the oleate molecules forming

a membrane plane around the Δβ-barrel and thereby shielding it from the hallucinated helices. The model is color coded according to the pLDDT values with very high confidence (pLDDT > 90) in blue, confident (90 > pLDDT > 70) in light blue, low confidence (70 > pLDDT > 50) in yellow, and very low confidence (pLDDT < 50) in orange. D) AF model of Intimin HA453. Not the change in the orientation of the extracellular domains in presence of the HA tag. Colors as in C). E) AF model of Intimin Δβ-strand. Colors as in C). F) AF model of Intimin Y448D. Colors as in C). G) AF model of Intimin Δloop4. Colors as in C). H) AF model of Intimin Δloop4. Colors as in C). I) AF model of Intimin Δlinker. Colors as in C). J) AF model of Intimin R434A. Colors as in C). K) AF model of Intimin Δα-helix. Colors as in C). M) AF model of Intimin ΔI382D. Colors as in C).

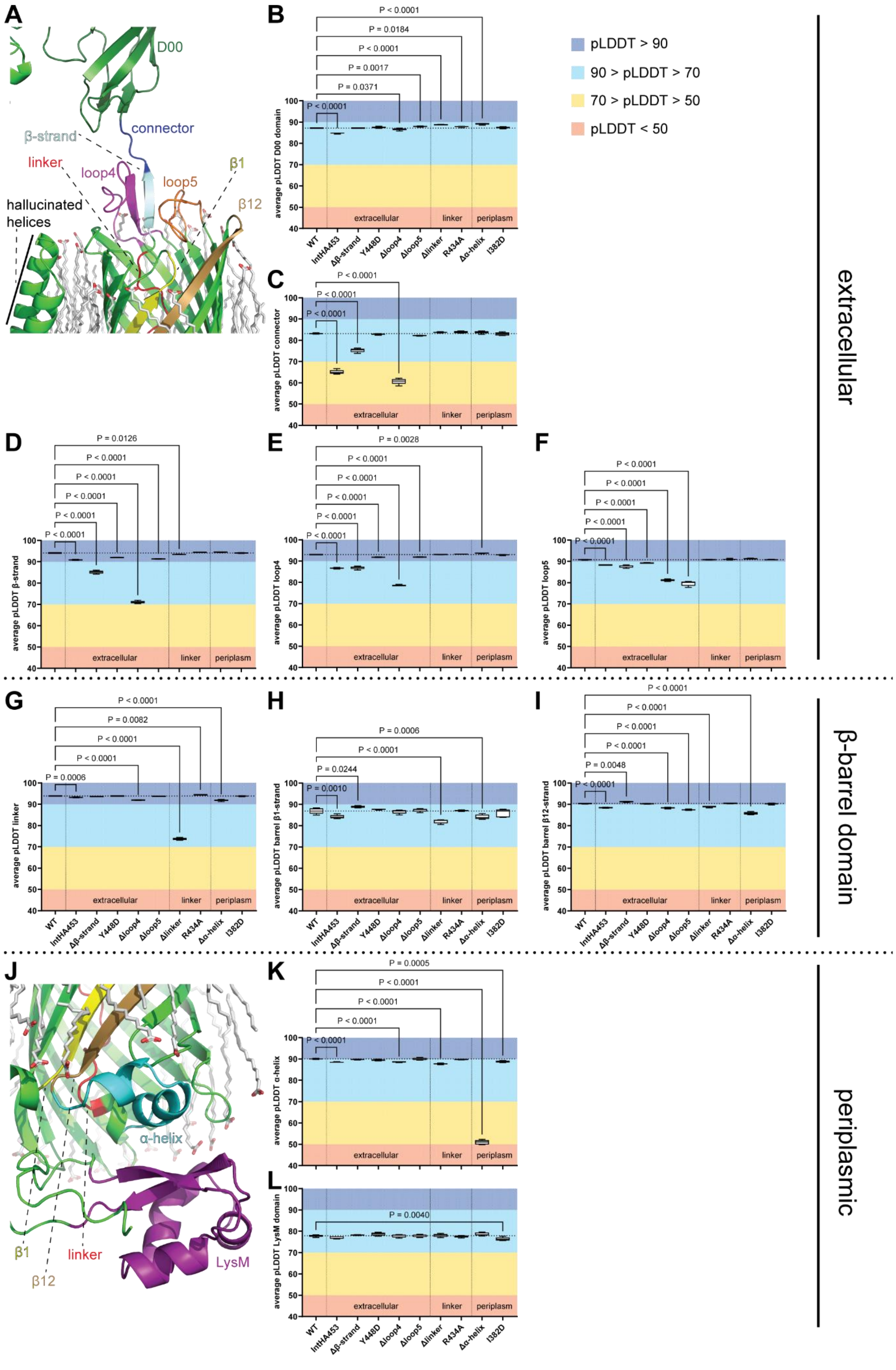
A
D00
β-strand
connector
linker
loop4
loop5
β1
β12
hallucinated helices
B
P < 0.0001
P = 0.0184
P < 0.0001
P = 0.0017
P = 0.0371
P < 0.0001
average pLDDT D00 domain
pLDDT > 90
90 > pLDDT > 70
70 > pLDDT > 50
pLDDT < 50
C
P < 0.0001
P < 0.0001
P < 0.0001
average pLDDT connector
D
P = 0.0126
P < 0.0001
P < 0.0001
P < 0.0001
P < 0.0001
P < 0.0001
average pLDDT β-strand
E
P = 0.0028
P < 0.0001
P < 0.0001
P < 0.0001
P < 0.0001
P < 0.0001
average pLDDT loop4
F
P < 0.0001
P < 0.0001
P < 0.0001
P < 0.0001
P < 0.0001
average pLDDT loop5
extracellular
G
P < 0.0001
P = 0.0082
P < 0.0001
P < 0.0001
P = 0.0006
average pLDDT linker
H
P = 0.0006
P < 0.0001
P = 0.0244
P = 0.0010
average pLDDT barrel β1-strand
I
P < 0.0001
P < 0.0001
P < 0.0001
P < 0.0001
P = 0.0048
P < 0.0001
average pLDDT barrel β12-strand
β-barrel domain
J
α-helix
β1
linker
β12
LysM
K
P = 0.0005
P < 0.0001
P < 0.0001
P < 0.0001
P < 0.0001
average pLDDT α-helix
L
P = 0.0040
average pLDDT LysM domain
periplasmic
extracellular
linker
periplasm
WT
IntHA453
Δβ-strand
Y448D
Δloop4
Δloop5
Δlinker
R434A
Δα-helix
I382D
100
90
80
70
60
50
40

Supplementary Figure 5. Mutations within the Intimin β-barrel influence the local folding. A) Extracellular side of the Intimin β-barrel. Intimin structure motifs are highlighted in the structure of full length Intimin as predicted by AF as in Figure 1 with the addition of the connector (blue) between the β-strand and D00 domain (dark green), the first β-strand (β1, yellow) and the last β-strand (β12, brown) of the Intimin β-barrel. Oleate molecules included in the AF predictions are shown in white. B) average pLDDT score of the extracellular D00 domain of the WT, the stalled IntHA453 variant, and several mutants (Δβ-strand, Y448D, Δloop4, Δloop5, Δlinker, R343A, Δα-helix, I382D) in the WT background. C) average pLDDT score of the extracellular connector N-terminal of the D00 domain of the WT, the stalled IntHA453 variant, and several mutants (see B)) in the WT background. D) average pLDDT score of the extracellular β-strand of the β-barrel of the WT, the stalled IntHA453 variant, and several mutants (see B)) in the WT background. E) average pLDDT score of the extracellular loop4 of the β-barrel of the WT, the stalled IntHA453 variant, and several mutants (see B)) in the WT background. F) average pLDDT score of the extracellular loop5 of the β-barrel of the WT, the stalled IntHA453 variant, and several mutants (see B)) in the WT background. G) average pLDDT score of the linker in the lumen of the β-barrel of the WT, the stalled IntHA453 variant, and several mutants (see B)) in the WT background. H) average pLDDT score of the first β-strand (β1) of the β-barrel of the WT, the stalled IntHA453 variant, and several mutants (see B)) in the WT background. I) average pLDDT score of the last β-strand (β12) of the β-barrel of the WT, the stalled IntHA453 variant, and several mutants (see B)) in the WT background. I) PEriplasmic side of the Intimin β-barrel. Intimin structure motifs are highlighted in the structure of full length Intimin as predicted by AF as in Figure 1 and panel A with the addition of the LysM domain (purple). K) average pLDDT score of the periplasmic α-helix of the WT, the stalled IntHA453 variant, and several mutants (see B)) in the WT background. M) average pLDDT score of the periplasmic LysM domain of the WT, the stalled IntHA453 variant, and several mutants (see A)) in the WT background. P-values were calculated by ordinary one-way ANOVA. For clarity, only P-values < 0.05 are shown. pLDDT bins are color coded according to default AF colors: Very high confidence (pLDDT > 90) in blue, confident (90 > pLDDT > 70) in light blue, low confidence (70 > pLDDT > 50) in yellow, and very low confidence (pLDDT < 50) in orange. The average pLDDT score of the WT region is indicated by a dashed horizontal line.

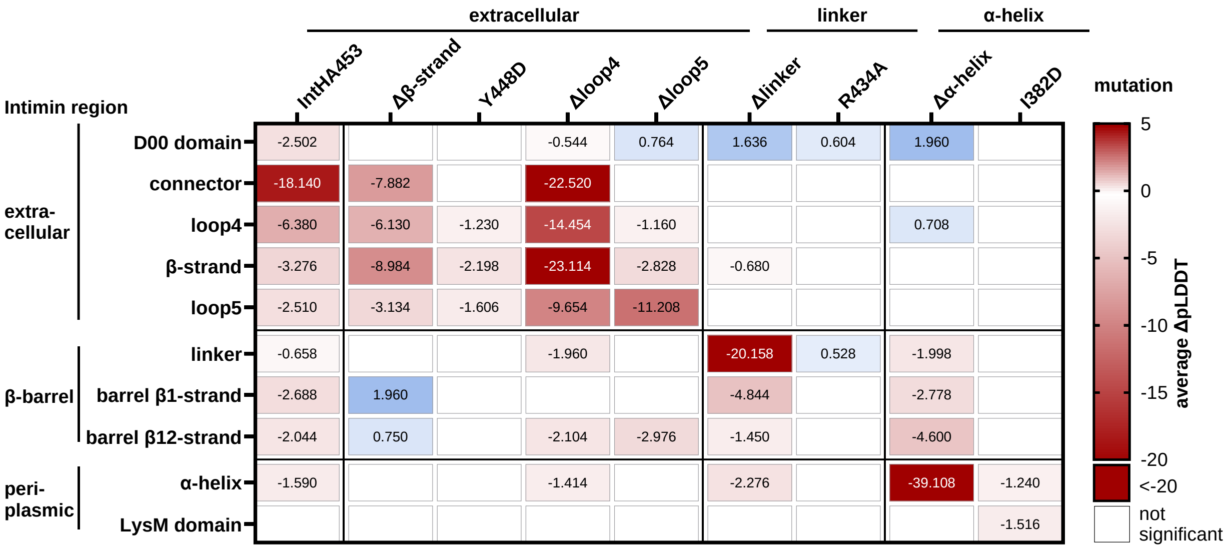

Supplementary Figure 6. Mutations within the Intimin β-barrel influence the region close to the mutation site. The average difference (average ΔpLDDT) of the pLDDT score for each Intimin region (left) is shown for each mutation of which AF models were created (horizontal). Positive differences (blue) indicate a stabilization of the predicted folding, whereas negative differences (red) indicate a destabilization. Only significant changes in comparison to the prediction of the WT protein ($P < 0.05$, Supplemenatry Figure 5) are blotted.

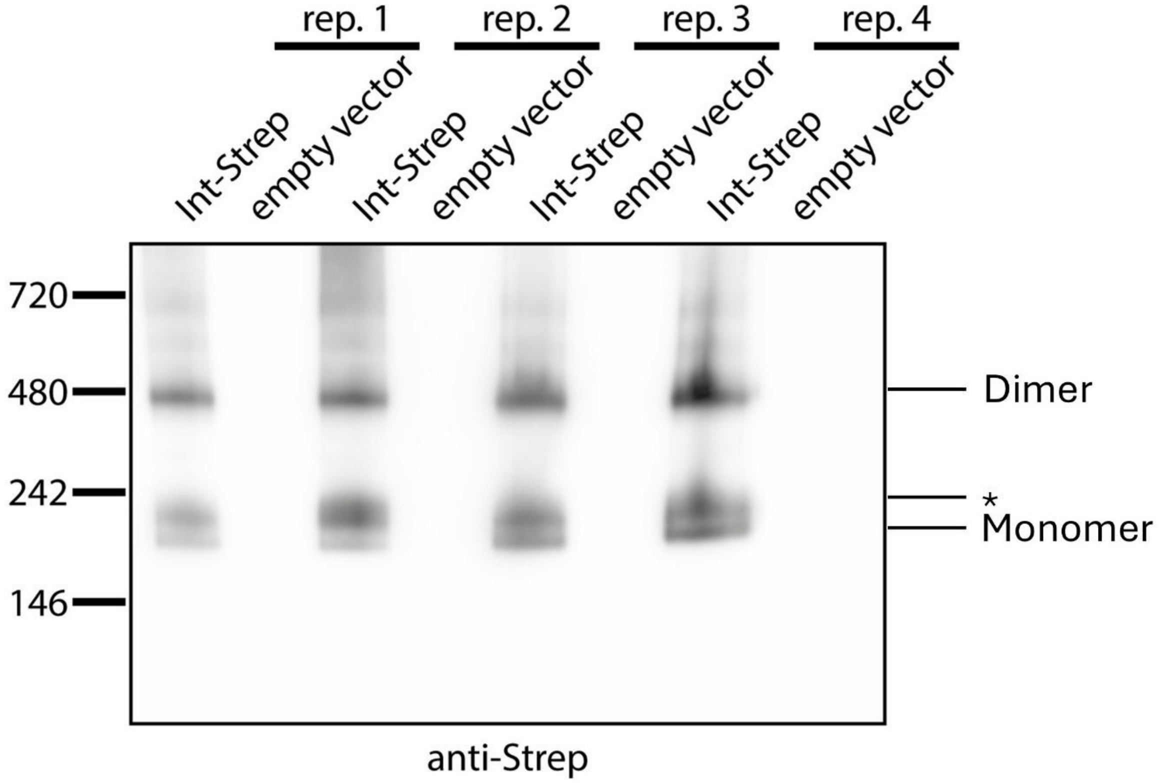

Supplementary Figure 7. Western blot of outer membrane samples with intimin variants using an anti-Strep tag primary antibody from four independent isolations. Samples were separated on a BN-PAGE (4-16% NativePAGE Bis-Tris Mini Protein Gel (Invitrogen)) and transferred onto a PVDF membrane. The molecular sizes of soluble proteins from the NativeMark™ Unstained Protein Standard (Invitrogen) is indicated. Of note, solubilization of membrane proteins usually leads to an increase in the observed size on a BN-PAGE due to the presence of membrane and detergent micelles surrounding the membrane inserted portions of the proteins. Three bands are seen using the anti-Strep antibody, which we interpret to be the intimin dimer (~480 kDa), the monomer (~200 kDa), and the additional band seen in denaturing PAGE (*). Intimin is known to form dimers [8], and in previous experiments, dimerisation dependent on the periplasmic domain leads to an equilibrium between monomers and dimers [9].

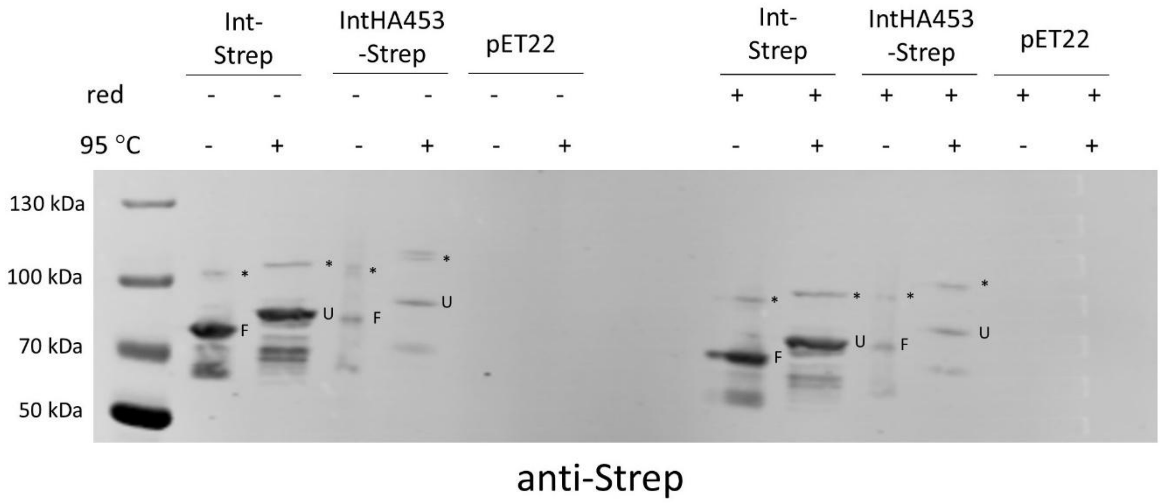

Supplementary Figure 8. The additional intimin band is not due to incorrect disulphide bond formation. Outer membrane samples with Int-Strep or IntHA453-Strep were split into two, and to one sample reducing agent (red, Thermo Fisher NuPAGE sample reducing agent) was added. The samples were then split again, and one half was incubated at room temperature while the other was heated for 10 minutes at 95 °C. The samples were then run in a 4-12% Novex gel before transferring to a nitrocellulose membrane and probing with an anti-Strep antibody. pET22 is the empty vector. Molecular weight standards are notated on the left. F = folded, U= unfolded, * = band of unknown origin.

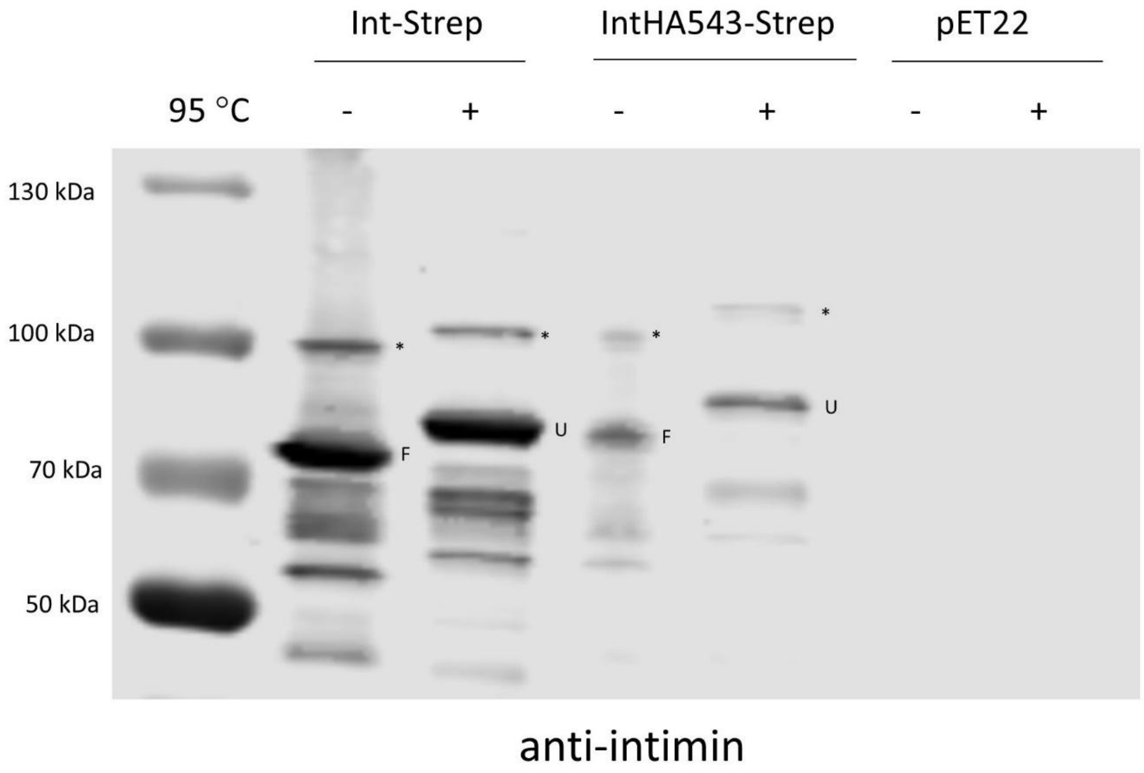

Supplementary Figure 9. Western blot of Int-Strep and IntHA453-Strep with an anti-intimin antibody recognising the C-terminus of intimin. Outer membrane samples with Int-Strep or IntHA453-Strep were split into two and one half was incubated at room temperature while the other was heated for 10 minutes at 95 °C. The samples were then run in a 4-12% Novex gel before transferring to a nitrocellulose membrane and probing with an anti-Intimin antibody [7]. pET22 is the empty vector. Molecular weight standards are notated on the left. F = folded, U= unfolded, * = band of unknown origin.

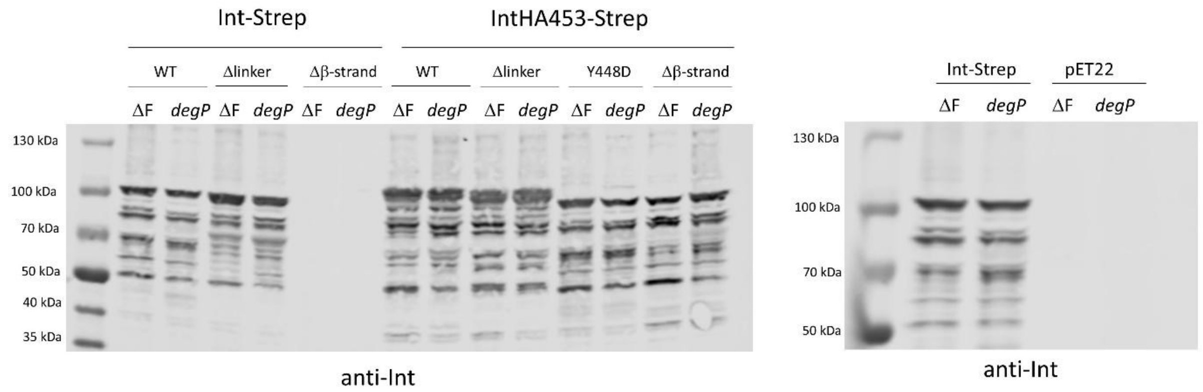

Supplementary Figure 10. Loss of protein in the Int-Strep Δβ-strand variant is not due to degradation by DegP. Whole-cell samples from autoinduced cultures of either BL21ΔF (ΔF) or BL21ΔF Δ*degP* (*degP*) expressing intimin variants were separated on a 4-12% gel and transferred to a nitrocellulose membrane before being probed with an anti-intimin antibody [7]. The blot on the left shows intimin variants in either the Int-Strep of the IntHA453-Strep backgrounds. The gel on the right is a control gel where pET22 is the empty vector. Molecular weight standards are notated on the left of the gels.

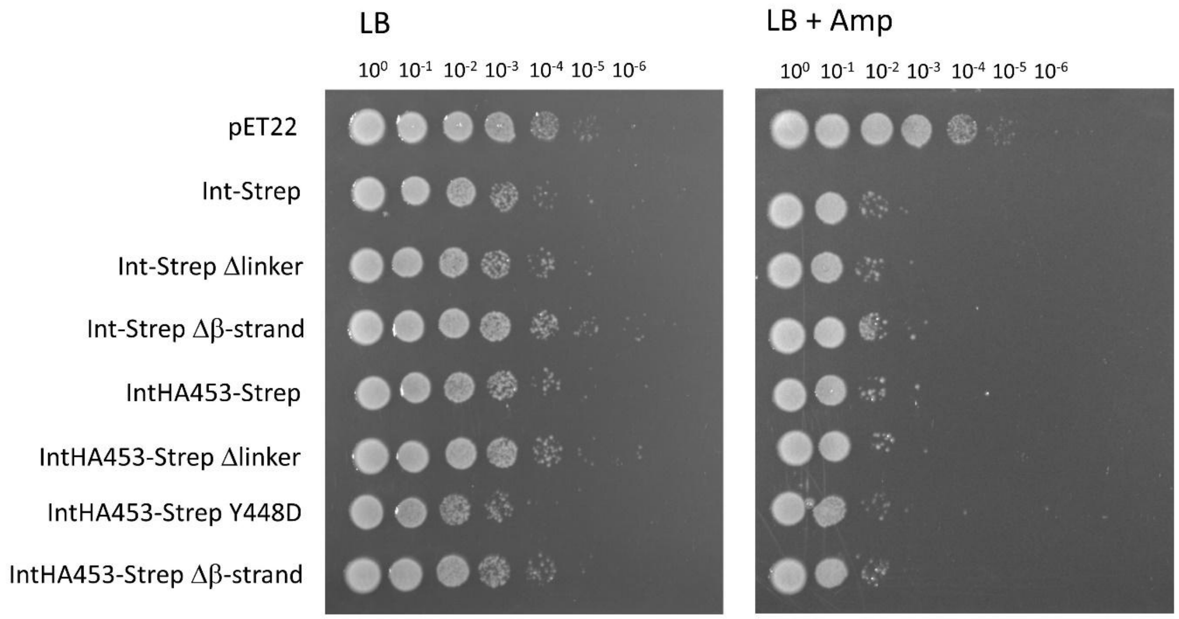

Supplementary Figure 11. Plasmid retention in cultures expressing intimin variants. Bacteria were cultured in autoinduction medium overnight and adjusted to the same optical density the following day. Serial dilutions were made and plated onto LB with no selection (left) or LB with ampicillin (right); bacteria lacking the plasmid will not grow on the ampicillin plate. Intimin variants show a similar level of plasmid loss in overnight cultures.

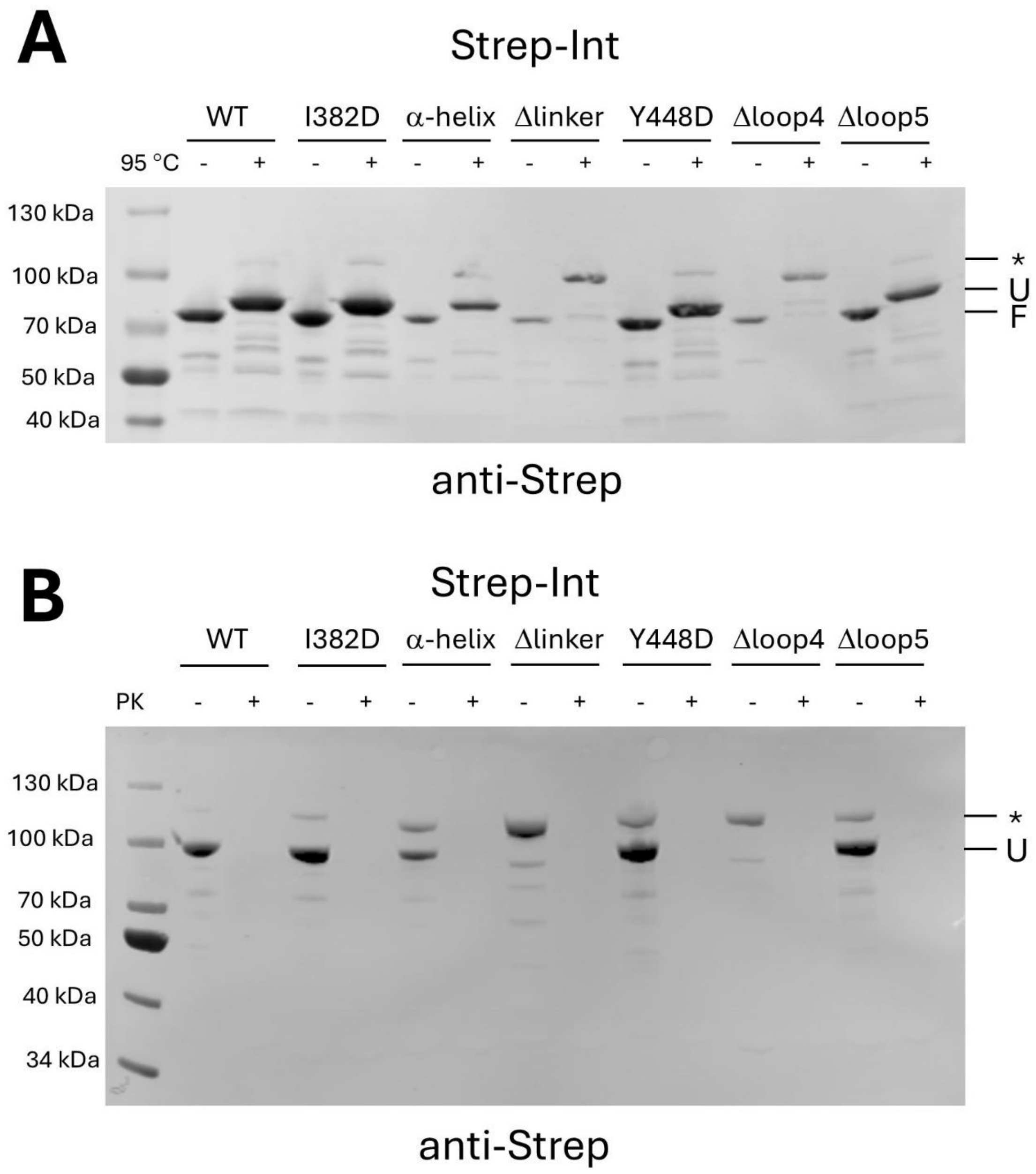

Supplementary Figure 12. Protease shaving of intimin mutants. For protease shaving assays, intimin variants in the WT background with an N-terminal StrepII tag were produced. A) Heat shift of Strep-Int variants, where OM samples from all variants display a heat shift when probed with anti-Strep after separation in a 4-12% SDS-PAGE gel when heated at 95 °C. B) Cells expressing Strep-Int variants were subjected to protease shaving with proteinase K (PK), after which OM samples were extracted, heated at 95°C and separated in a 4-20% gel, transferred to a nitrocellulose membrane and probed with an anti-Strep antibody. All variants lost the signal after protease treatment. Molecular weight standards are notated on the left. F = folded, U= unfolded, * = band of unknown origin.

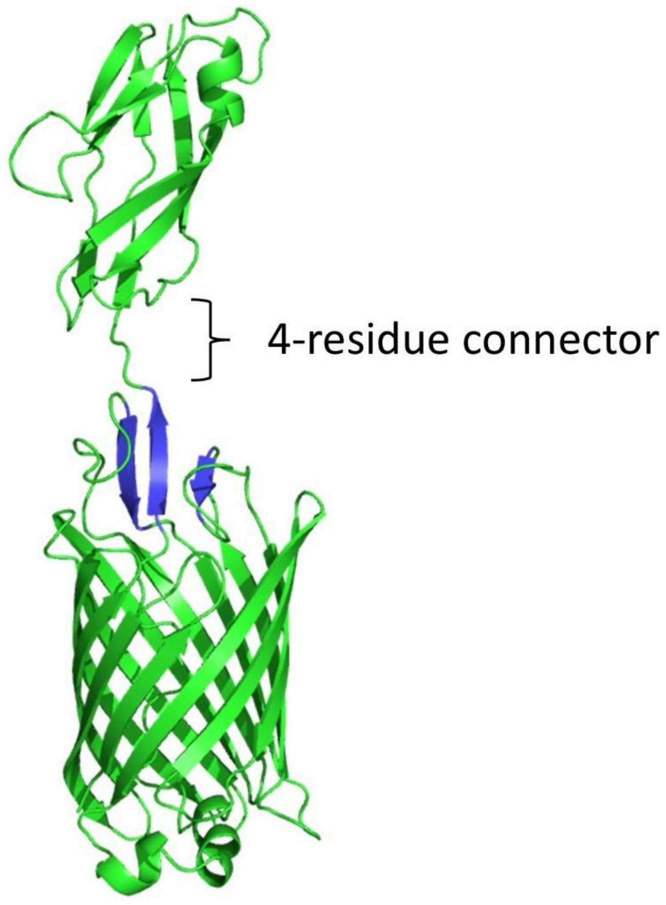

Supplementary Figure 13. Alphafold model of the intimin β-barrel domain and D00 domain. The model shows a 4-residue extended connector element between the β-sheet at the extracellular face of the β-barrel (in blue) and the Ig-like D00 domain.

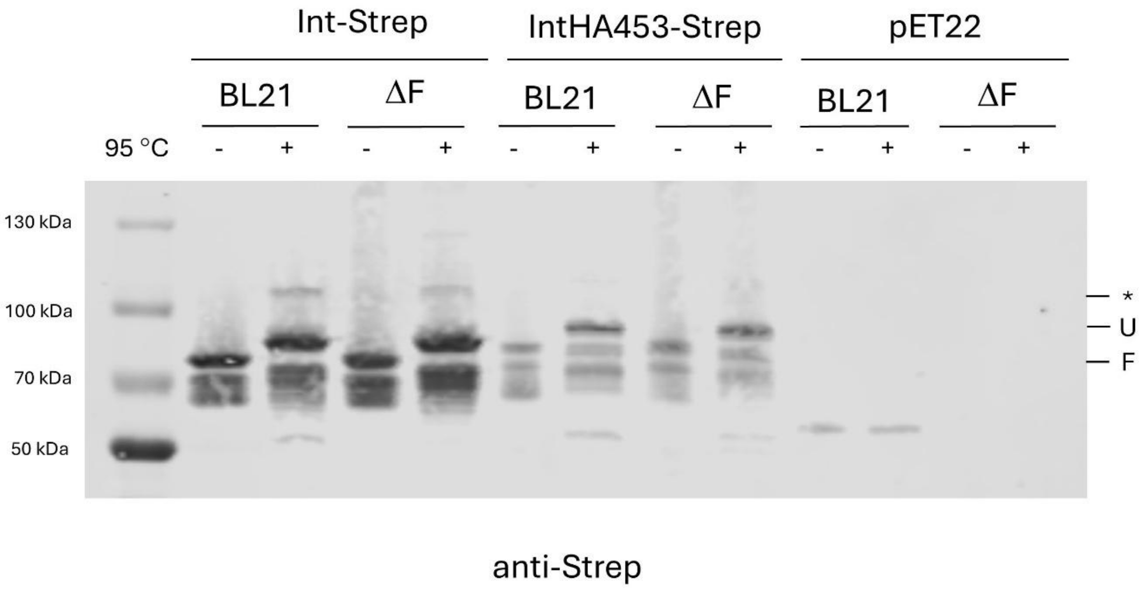

Supplementary Figure 14. BL21Gold(DE3) and BL21ΔF produce Int-Strep and IntHA453-Strep at similar levels. OM samples from the two strains expressing intimin variants or the empty vector (pET22) were split in two and one half was heated to 95°C for 10 minutes, while the other half was kept at room temperature. The samples were then separated in a 4-12% gel, transferred to a nitrocellulose membrane and probed with an anti-Strep antibody. Molecular weight standards are notated on the left. F = folded, U= unfolded, * = band of unknown origin.

Supplementary references:

1 Abramson, J., Adler, J., Dunger, J., Evans, R., Green, T., Pritzel, A., Ronneberger, O., Willmore, L., Ballard, A. J., Bambrick, J., Bodenstein, S. W., Evans, D. A., Hung, C., O'neill, M., Reiman, D., Tunyasuvunakool, K., Wu, Z., Žemgulytė, A., Arvaniti, E., Beattie, C., Bertolli, O., Bridgland, A., Cherepanov, A., Congreve, M., Cowen-Rivers, A. I., Cowie, A., Figurnov, M., Fuchs, F. B., Gladman, H., Jain, R., Khan, Y. A., Low, C. M. R., Perlin, K., Potapenko, A., Savy, P., Singh, S., Stecula, A., Thillaisundaram, A., Tong, C., Yakneen, S., Zhong, E. D., Zielinski, M., Žídek, A., Bapst, V., Kohli, P., Jaderberg, M., Hassabis, D. and Jumper, J. M. (2024) Accurate structure prediction of biomolecular interactions with AlphaFold 3. Nature. **630**, 493–500. doi:10.1038/s41586-024-07487-w

2 Hallgren, J., Tsirigos, K. D., Pedersen, M. D., Almagro Armenteros, J. J., Marcatili, P., Nielsen, H., Krogh, A. and Winther, O. (2022) DeepTMHMM predicts alpha and beta transmembrane proteins using deep neural networks. bioRxiv. doi:10.1101/2022.04.08.487609

3 Mészáros, B., Erdos, G. and Dosztányi, Z. (2018) IUPred2A: Context-dependent prediction of protein disorder as a function of redox state and protein binding. Nucleic Acids Res. **46**, W329–W337. doi:10.1093/nar/gky384

4 Erdős, G. and Dosztányi, Z. (2020) Analyzing protein disorder with IUPred2A. Curr Protoc Bioinformatics. **70**, e99–n/a. doi:10.1002/cpbi.99

5 Leo, J. C., Oberhettinger, P., Yoshimoto, S., Udatha, D. B., Morth, J. P., Schütz, M., Hori, K. and Linke, D. (2016) Secretion of the intimin passenger domain is driven by protein folding. J Biol Chem. **291**, 20096–20112. doi:10.1074/jbc.M116.731497 [doi]

6 Oberhettinger, P., Leo, J. C., Linke, D., Autenrieth, I. B. and Schütz, M. S. (2015) The inverse autotransporter intimin exports its passenger domain via a hairpin intermediate. J Biol Chem. **290**, 1837–1849. doi:10.1074/jbc.M114.604769

7 Masters, M. R., Mahmoud, A. H. and Lill, M. A. (2025) Investigating whether deep learning models for co-folding learn the physics of protein-ligand interactions. Nat Commun. **16**, 8854–12. doi:10.1038/s41467-025-63947-5

8 Touzé, T., Hayward, R. D., Eswaran, J., Leong, J. M. and Koronakis, V. (2004) Self-association of EPEC intimin mediated by the β-barrel-containing anchor domain: A role in clustering of the tir receptor. Mol. Microbiol. **51**, 73–87. doi:10.1046/j.1365-2958.2003.03830.x

9 Leo, J. C., Oberhettinger, P., Chaubey, M., Schütz, M., Kühner, D., Bertsche, U., Schwarz, H., Gotz, F., Autenrieth, I. B., Coles, M. and Linke, D. (2015) The intimin periplasmic domain mediates dimerisation and binding to peptidoglycan. Mol Microbiol. **95**, 80–100. doi:10.1111/mmi.12840

Uncropped western blot images

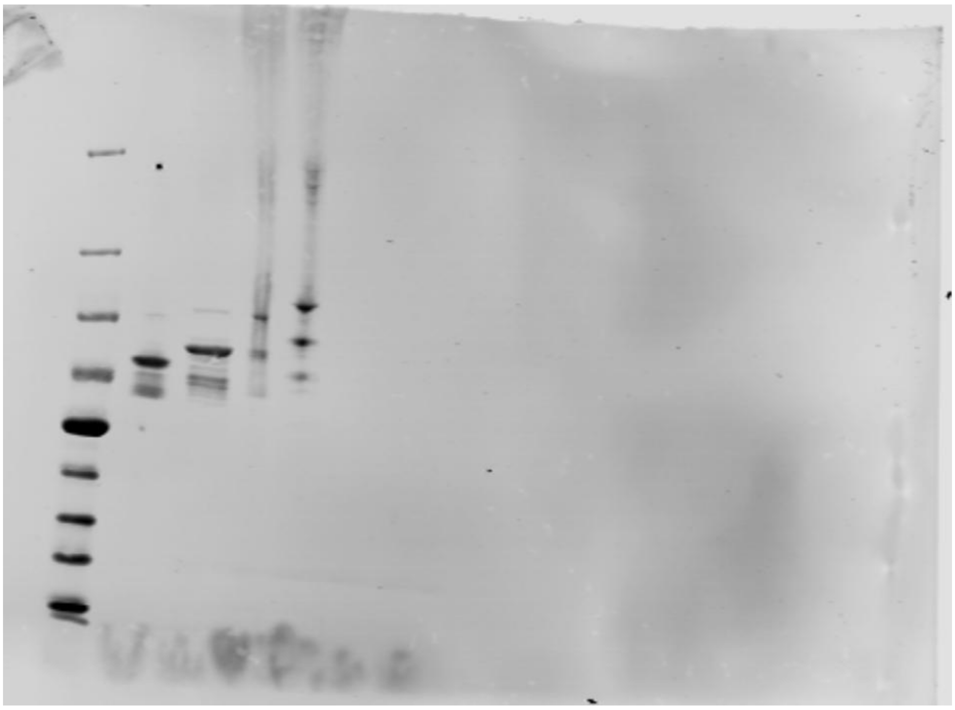

Figure 3

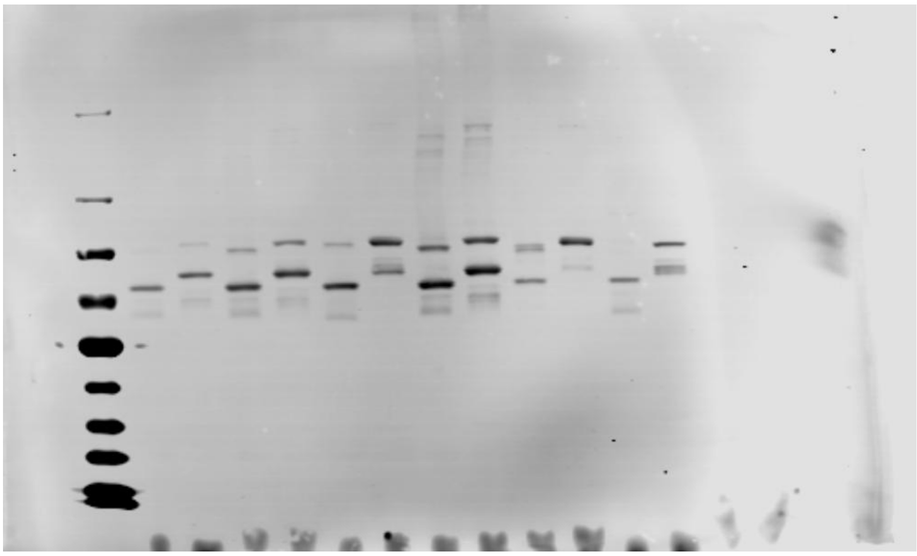

Figure 4 (upper gel)

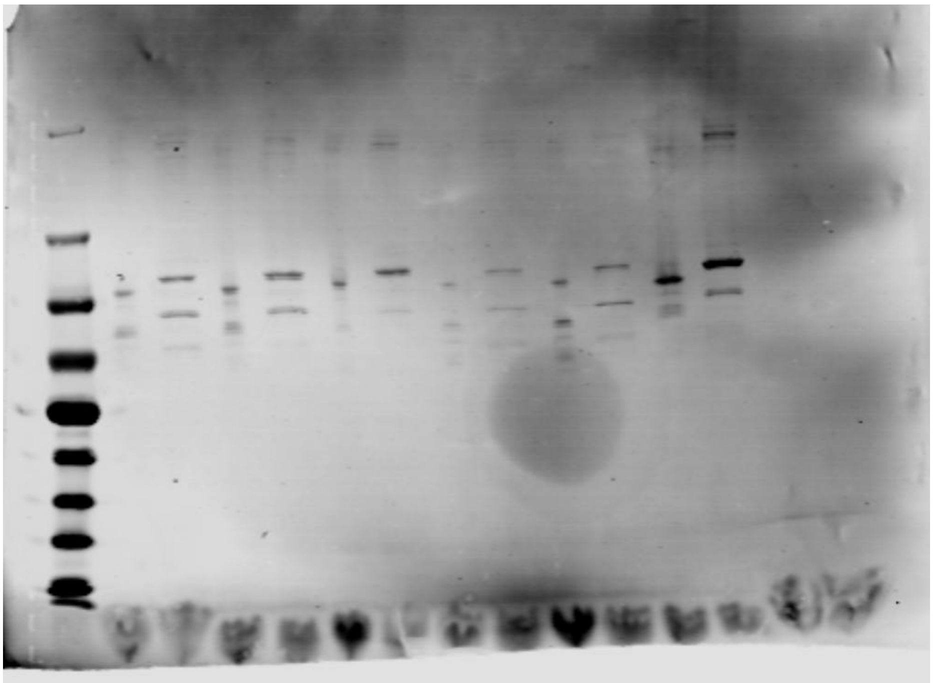

Figure 4 (lower gel)

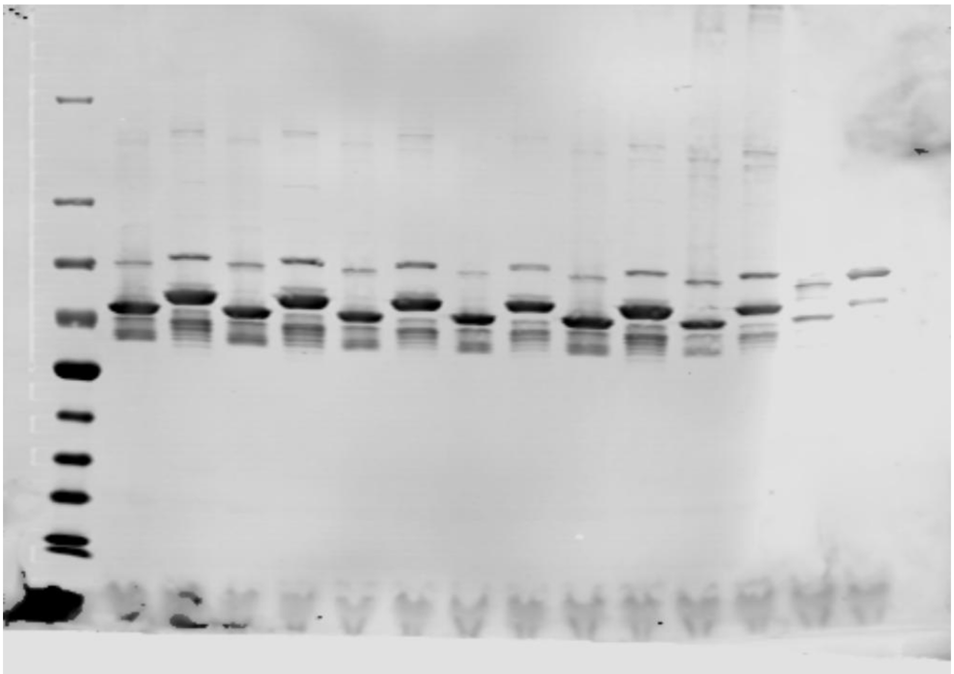

Figure 5 (upper gel)

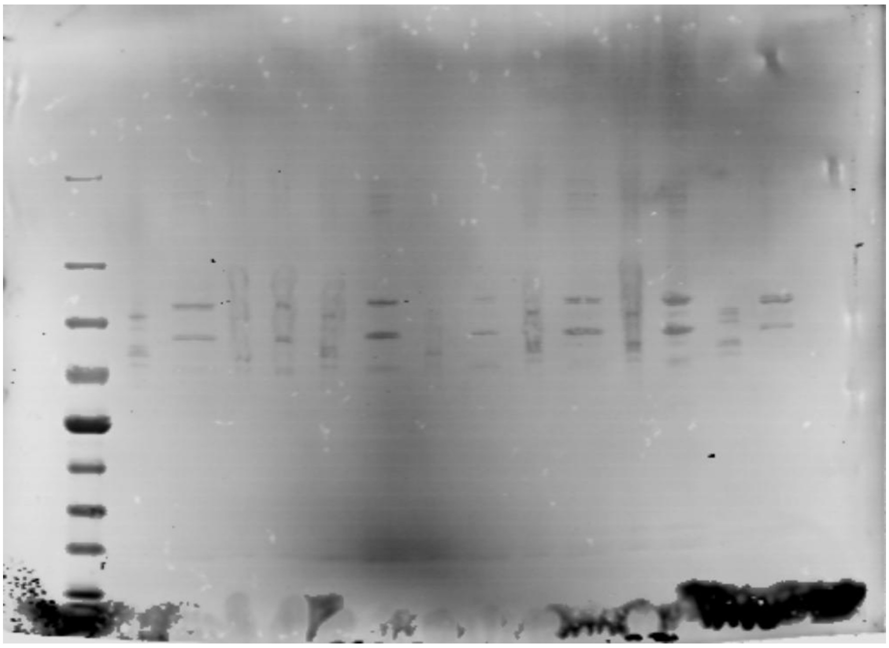

Figure 5 (lower gel)

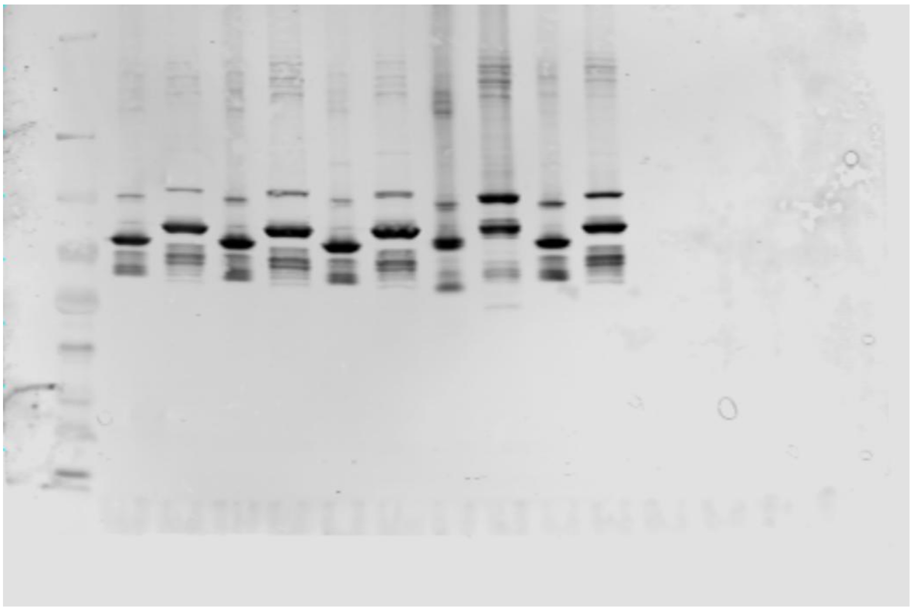

Figure 6 (upper gel)

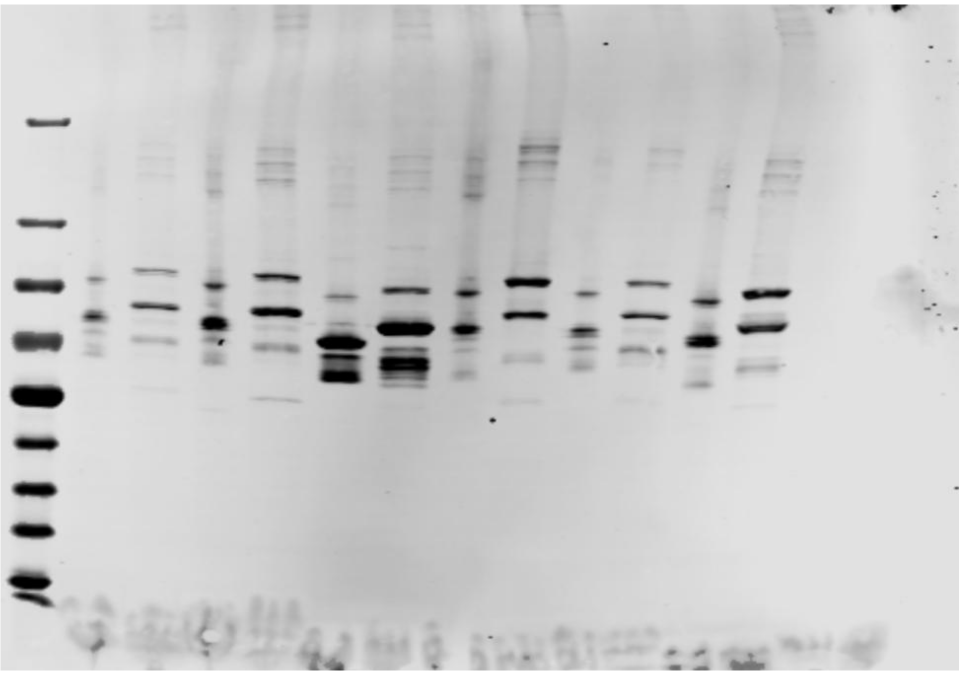

Figure 6 (lower gel)

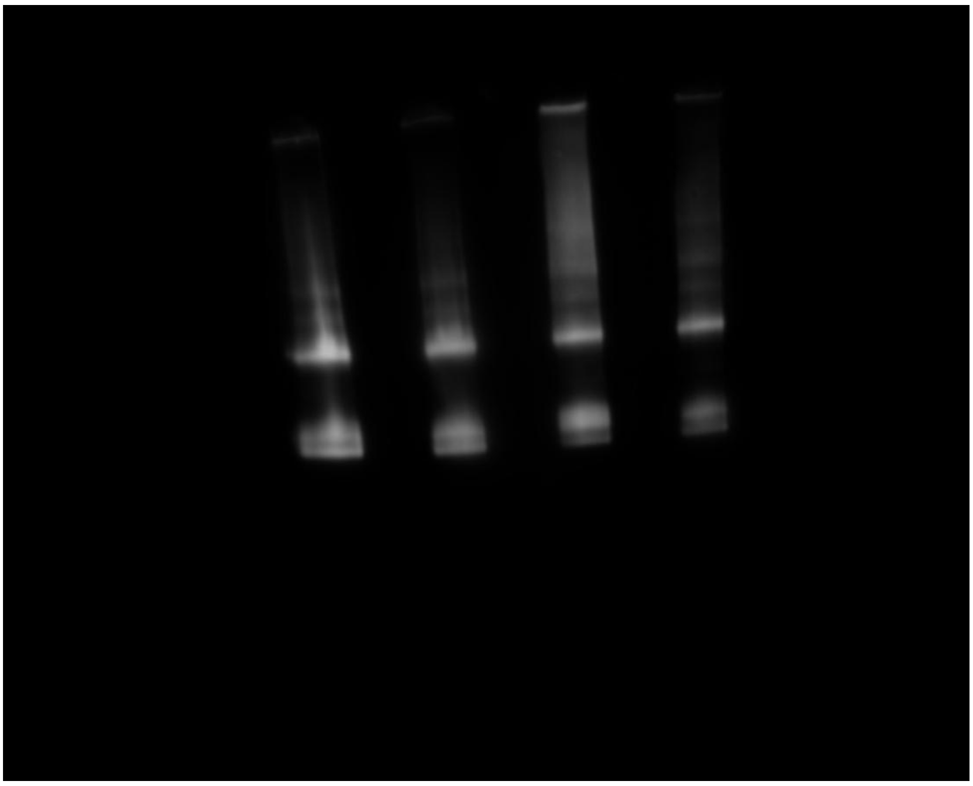

Supplementary Figure 7

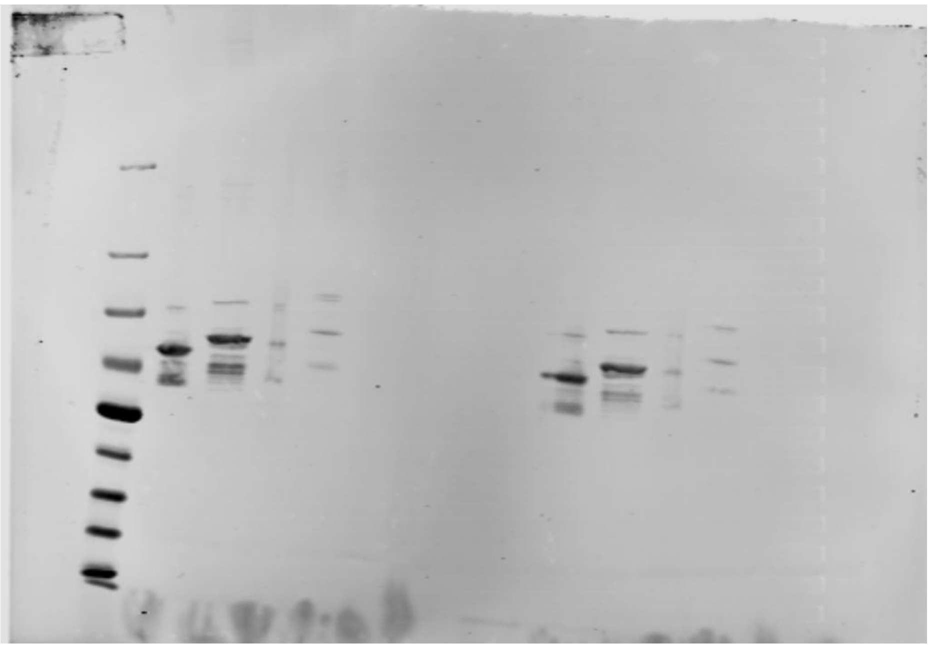

Supplementary Figure 8

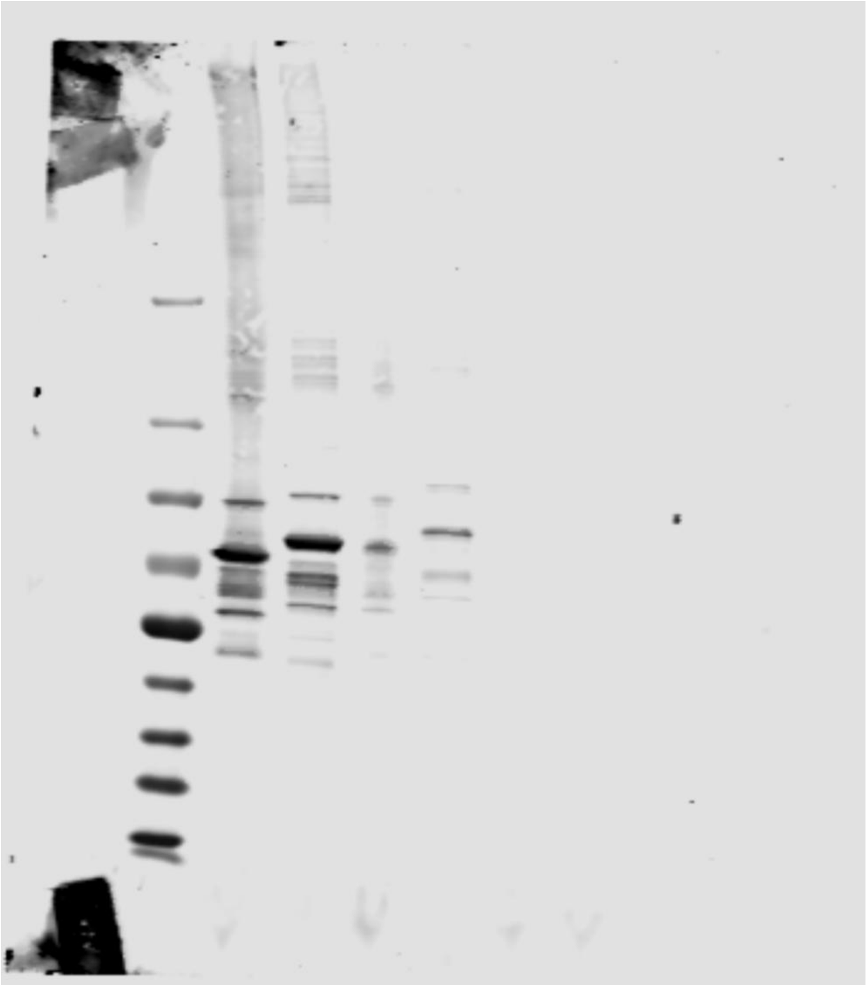

Supplementary Figure 9

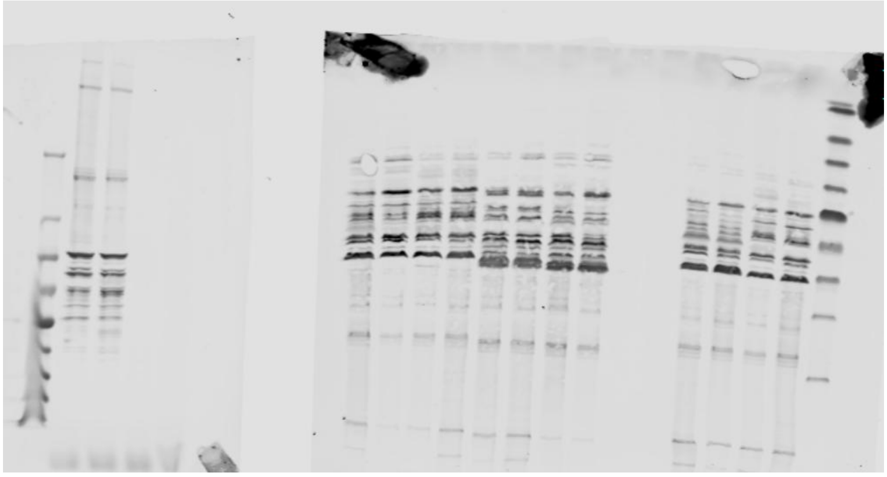

Supplementary Figure 10

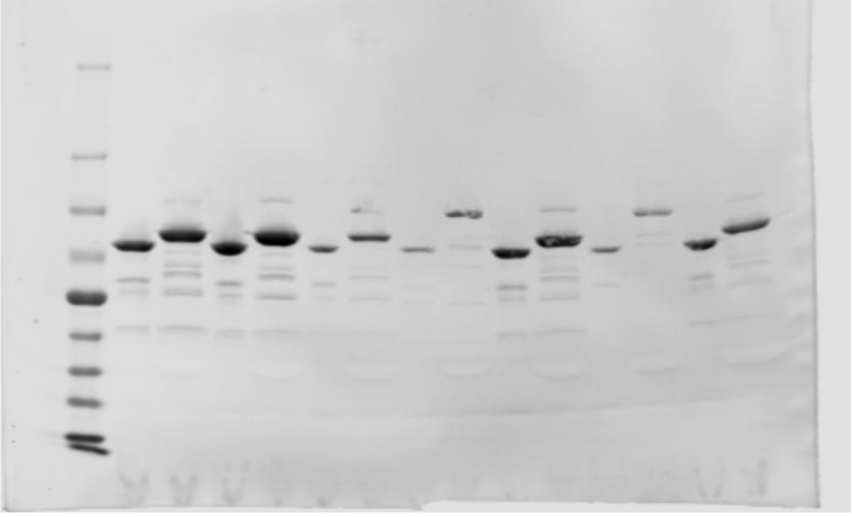

Supplementary Figure 12 (upper gel)

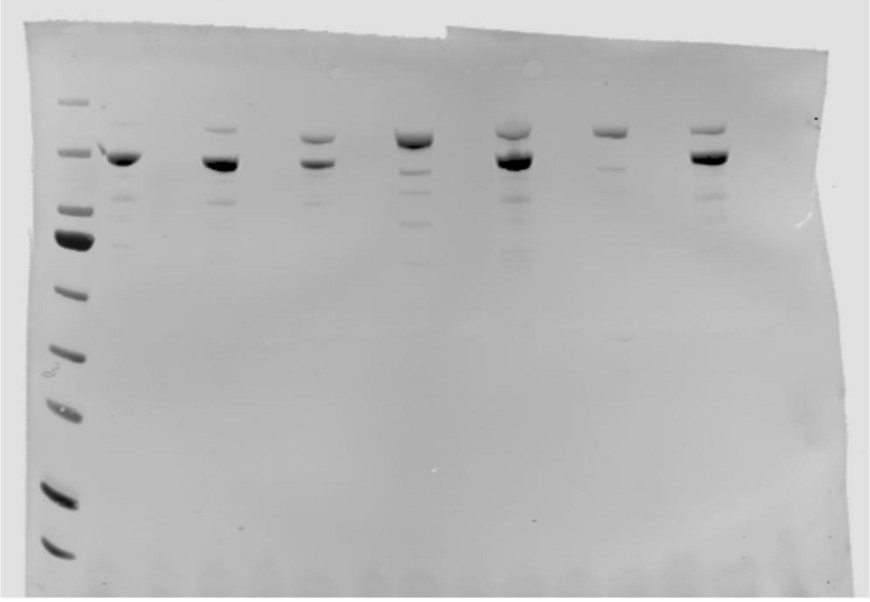

Supplementary Figure 12 (lower gel)

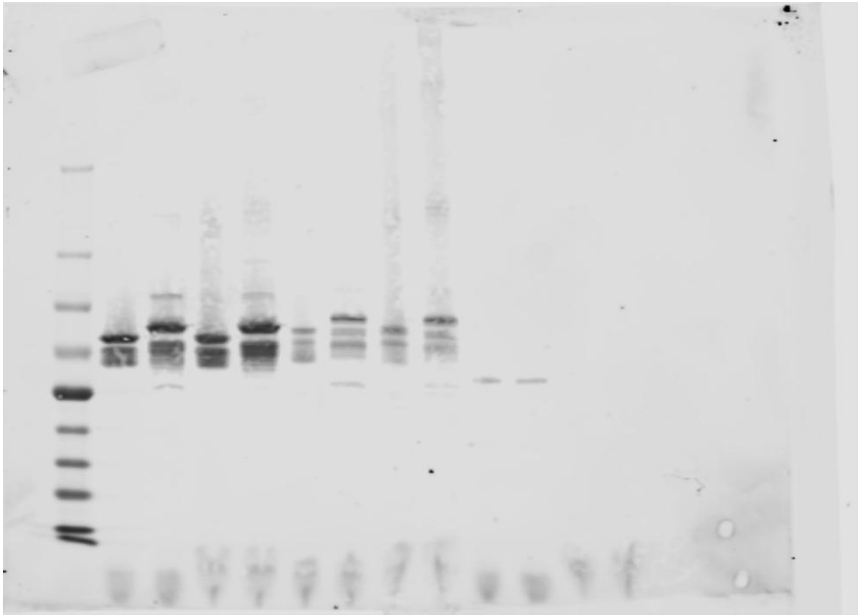

Supplementary Figure 14
